# Sensor Control in Anti-Submarine Warfare—A Digital Twin and Random Finite Sets Based Approach

**DOI:** 10.3390/e21080767

**Published:** 2019-08-06

**Authors:** Peng Wang, Mei Yang, Yong Peng, Jiancheng Zhu, Rusheng Ju, Quanjun Yin

**Affiliations:** College of Systems Engineering, National University of Defense Technology, Changsha 410073, China

**Keywords:** digital twin, anti-submarine warfare (ASW), data assimilation, online sensor control, random finite set (RFS), sequential Monte Carlo (SMC), Bayesian inference

## Abstract

Since the submarine has become the major threat to maritime security, there is an urgent need to find a more efficient method of anti-submarine warfare (ASW). The digital twin theory is one of the most outstanding information technologies, and has been quite popular in recent years. The most influential change produced by digital twin is the ability to enable real-time dynamic interactions between the simulation world and the real world. Digital twin can be regarded as a paradigm by means of which selected online measurements are dynamically assimilated into the simulation world, with the running simulation model guiding the real world adaptively in reverse. By combining digital twin theory and random finite sets (RFSs) closely, a new framework of sensor control in ASW is proposed. Two key algorithms are proposed for supporting the digital twin-based framework. First, the RFS-based data-assimilation algorithm is proposed for online assimilating the sequence of real-time measurements with detection uncertainty, data association uncertainty, noise, and clutters. Second, the computation of the reward function by using the results of the proposed data-assimilation algorithm is introduced to find the optimal control action. The results of three groups of experiments successfully verify the feasibility and effectiveness of the proposed approach.

## 1. Introduction

Submarines are the main combat forces of modern maritime warfare, and the major threats to maritime security. Anti-submarine warfare (ASW) is a type of warfare that depends on surface warships, aircraft, or submarines to fight against enemy submarines. The key of ASW is to quickly identify and localize as many enemy submarines as possible. Sensor control is the key technology for the victory in ASW, so we focus on the innovation of the online sensor control method. Many works have been done to apply simulation-based approaches in naval warfare research, but there are quite a few effective methods for combining simulation technologies with the real ASW in real time. In this paper, we study how to control the sensor of anti-submarine ships in ASW by employing simulation theory, random finite set (RFS) theory, and digital twin theory.

The sensor control problem is also known as sensor management problem. The sensor equipped on the anti-submarine ship can perform many different actions including moving to designated areas, searching in certain directions, etc. In real ASW, the anti-submarine ship usually takes tactical actions to estimate the accurate distance and guarantee the observability [1,2,3]. Different actions have different effectiveness; some actions will be effective for sensing the submarine, while some others not. Here the goal of sensor control is to ensure maximum efficiency of sensors and provide more accurate measurements to the simulation system. Finding the optimal control action is an urgent need in practical application. In this paper, the objective of sensor control in ASW is choosing control actions online so that the utility of sensors is maximized. Here, sensor control means sequential decision making, where each control decision improves the utility of sensors and helps to win the victory in ASW.

In [4], a practical implementation of sensor control based on Kullback–Leibler divergence is proposed for the first time. In [5], the Rényi divergence is used for deriving the reward function. In [6], Kullback–Leibler discrimination is used as the reward function for sensor control, but the authors did not provide the implementation method. In [7], the posterior expected number of targets (PENT)-based reward function is given. PENT has been successfully applied in some applications [8,9]. In [10], the Cauchy-Schwarz divergence is used, and the analytical solution is also derived. These works all depend on the expected divergence or information gain between the predicted and posterior densities for choosing the optional control action. Their major limitations are the problems of significant computational cost, and too little use of simulation methods. The simulation methods are the key for taking more complex dynamic application scenarios into consideration. It is worth mentioning that some divergences usually fail to be analytically computed.

RFS-based Bayesian inference has an advantage in dealing with situations where the number of targets and received detections are random, and the targets’ states and detections are also random. The RFS-based Bayesian inference can successfully overcome the limitations of the conventional vector-based Bayesian inference, and is gradually applied to the sensor control problem. In [11], the Rényi divergence and RFS-based reward function is derived, and the probability hypothesis density-based filter is adopted for implementing the sensor control method. In [12], the RFS and Rényi divergence-based reward function is derived, and the CB-MemBer filter-based implementation of the sensor control method is proposed. In [13], the RFS-based analytical solution for Cauchy-Schwarz divergence is derived. In [14], the Cauchy-Schwarz divergence and generalized labeled multi-Bernoulli filter-based sensor control method is derived. In [15], a labeled multi-Bernoulli filter and Cauchy-Schwarz divergence-based constrained sensor control method is proposed. In [16], the authors control the sensors by using the expected risk reduction between the multi-target predicted and updated densities.

The framework of partially observed Markov decision process (POMDP) is used to study the problem of making the next decision by using the past decisions and past observations [17]. The current information state, a set of admissible sensor actions and the reward function associated with each action are the main elements of POMDP. The above-mentioned works all adopt the framework of POMDP. POMDP is widely used in robot control, moving target searching, object identification, and so on. POMDP depends on the assumption that the states of the studied system are determined by the Markov process. POMDP is lack of use of the prediction and evaluation ability of simulation theory, so it fails to take many complex simulation scenarios into consideration. Since it focuses on the basic mathematical theory, its ability to apply new information technologies is also weak. POMDP depends on the connection of the virtual space and the physical space, but it fails to tell us how to connect these different spaces. In this paper, we propose to use a more promising framework to solve the problem of sensor control in ASW.

Recently, digital twin is seen as the best way to enable the interoperability and integration between the real and simulated worlds [18]. At present, there is no formal and consistent definition of digital twin, and there are many different versions of definitions of digital twin. Different industries and application fields have different definition perspectives and methods. However, the core ideas of all definitions are similar. Grieves defines the digital twin as “a set of virtual information constructs that fully describes a potential or actual physical manufactured product from the micro atomic level to the macro geometrical level” [19,20,21]. Shafto et al. define the digital twin as “an integrated multiphysics, multiscale simulation of a vehicle or system that uses the best available physical models, sensor updates, fleet history, etc., to mirror the life of its corresponding flying twin” [22,23]. These definitions are the most cited, but for the research of sensor control in ASW, they seem to be unsuitable. In this paper, digital twin depends on creating the simulation models for related real entities in the digital way to simulate their behaviors, serving as a bridge between the real and the simulated systems [24,25]. The adaptability of the digital twin-based framework to ASW is reflected in the following aspects:

1. Digital twin breaks the barriers between the real ASW and the simulated ASW, and enables real-time communication in both directions.
2. Digital twin can make full use of the prediction, evaluation, and analysis ability of simulation system to evaluate available courses of actions for sensor control.
3. Digital twin paves a way for the cyber-physical integration of ASW, which is an important bottleneck to enable intelligent and adaptive decision making.
4. Digital twin can enable the integrated application of new-generation information technologies, such as internet of things (IoT), 5G, AI, cloud, edge computing, and so on.
5. Digital twin can take full advantage of POMDP and the simulation-based approach to support more complex application scenarios.

The backbone technology of digital twin is simulation for prediction, evaluation and analysis [24]. The simulation-based prediction with high confidence is the fundamental function of digital twin. The vision of the digital twin itself refers to a comprehensive functional description, evaluation, and prediction together with all available operational data of an entity, target, or system, which includes more or less all information which could be useful in all the current and subsequent phases [26]. The digital twin in ASW is not only used to describe and predict the behaviors of the submarine in the real ASW, but also to derive and evaluate solutions and course of actions (COAs) relevant to the real ASW.

The digital twin theory has been quite popular in recent years, but most of the research related to it only focuses on the theoretical research and requirement analysis. The practical application and implementation of digital twin has rarely been mentioned. This paper is the first attempt to apply and implement the digital twin theory. Simulation models for ASW are very complicated now, but still fail to describe the real ASW accurately. The main reason is the separation of simulation system and the real system, and the second reason is the failure to simultaneously integrate online measurements into the running simulation models.

In the proposed digital twin-based framework of sensor control in ASW, the online measurements are dynamically assimilated into the running simulation models, and the running simulation models guide the real ASW process in reverse. The intuitive application of digital twin for ASW is to obtain estimated states or parameters of the real ASW system by combining the real-time measurements with a simulation model [27]. Since real-time measurements can indicate the latest updated states of the real system, we focus on the problem of effectively assimilating continuous streams of data into running simulation models. At the same time, we have also studied the computation of reward function by using the results of the proposed data-assimilation algorithm.

The rest of the paper is structured as follows. We give the digital twin and RFS-based framework of sensor control in ASW in Section 2. The proposed RFS-based models for digital twin are introduced in Section 3. The RFS-based data-assimilation algorithm is detailed in Section 4. Section 5 describes the computation of the reward function by using the results of the proposed data-assimilation algorithm. Experimental results are detailed in Section 6, and the conclusions are given in Section 7.

## 2. Digital Twin and RFS-Based Framework of Online Sensor Control

**Figure 1:**
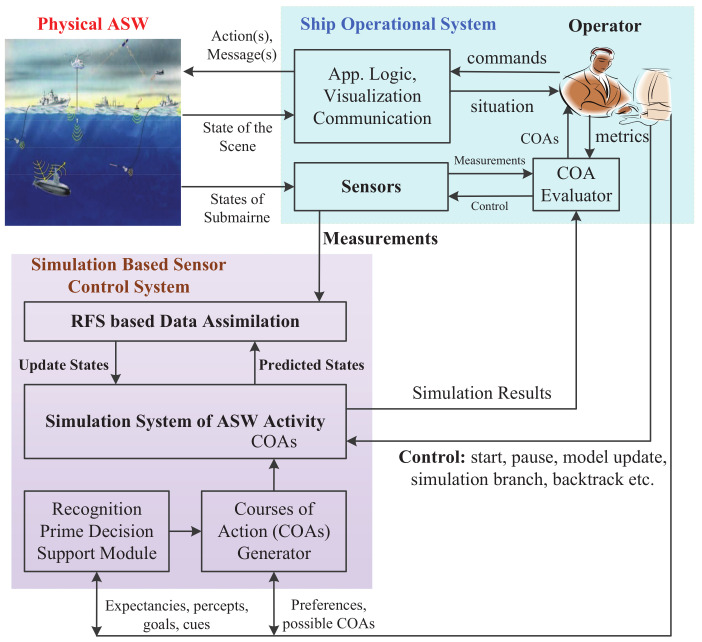
The operational view of the digital twin-based framework of sensor control in ASW.

As it is shown in Figure 1, the digital twin in ASW is mainly used for decision making. The digital twin can be regarded as a virtual equivalent or dynamic digital representation of the real ASW [28]. The simulated ASW in this framework is used to predict the emergent behaviors in the real ASW, evaluate the COAs and choose the best one for the operator. The simulated ASW evolves with the real ASW along the whole life cycle and integrates the currently available and commonly required data and knowledge. We can get the prediction, evaluation, and analysis of an enemy submarine by means of precise simulations. Digital twin can assist in ensuring information continuity throughout the whole operation, sensor control, and system behavior predictions in ASW based on simulations.

**Figure 2:**
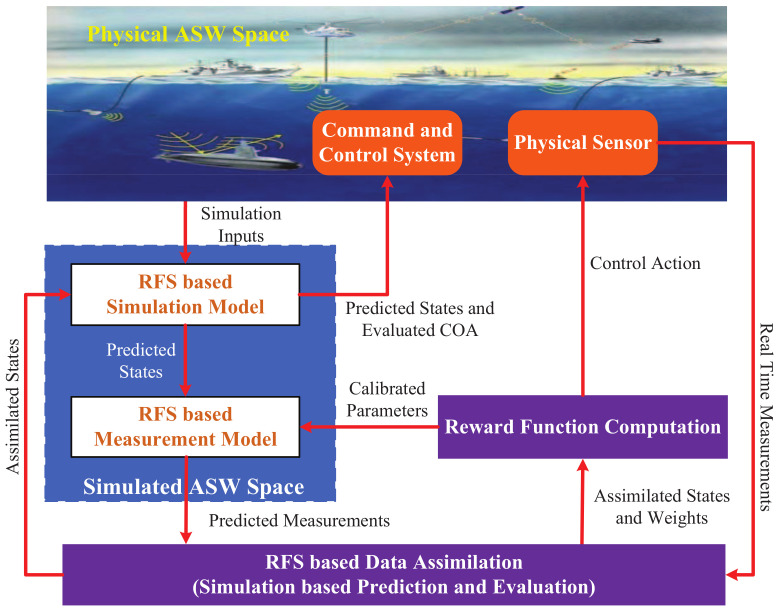
The technical view of the digital twin and RFS-based framework of online sensor control.

To improve the coordination between the simulation system and the real system for sensor control in ASW, we propose the digital twin-based framework of online sensor control by incorporating RFS theory. The technical view of the proposed framework is given in Figure 2. Since there are two constituent objects (simulated space and physical space) in digital twin, in this paper, we propose two corresponding technologies to support the implementation of the digital twin-based framework: one is the RFS-based data-assimilation algorithm for assimilating real-time measurements into the running simulation model, and the other one is the computation of the reward function by using the results of the proposed data-assimilation algorithm for finding the optimal control action.

Just as it is shown in Figure 2, the proposed feed-back control loop incorporates real-time measurements into the running simulation model while dynamically managing the physical sensors to refine measurements. The physical ASW space provides the simulation inputs to the simulation model, and it also provides the real states to the physical sensors.

The RFS-based simulation model provides the predicted states to the RFS-based measurement model. The simulation model helps to analyze potential alternative solutions for the anti-submarine ship, and evaluate the impact of possible control actions for the online sensor control method. The RFS-based measurement model characterizes the behaviors of the physical sensor on the anti-submarine ship. It uses the predicted states outputted by the RFS-based simulation model to generate the predicted measurements and provides them to the data-assimilation process. The RFS-based simulation model and measurement model are the main elements of the simulated ASW space.

The digital twin is supported by data-assimilation algorithm which can incorporate real-time measurements into the running simulation models for more accurate prediction of the physical ASW system, and it can also evaluate the COAs [29]. RFS-based data-assimilation process is the foundation of digital twin and is in charge of fusing real-time measurements to estimate the states of the physical ASW space [30]. There are two tasks for the data-assimilation process, the first is to dynamically update the current simulation states of the physical ASW space and provide the updated states to the simulation model for subsequent simulation running; the second is to provide the updated states and corresponding weights to the reward function computation module for computing the reward function.

## 3. RFS-Based Modeling of the Simulated ASW

The simulated ASW in digital twin depends on two kinds of models to describe the physical ASW: one is the Markov transition density-based simulation model; the other one is the measurement likelihood-based measurement model. Here we use the RFS-based simulation model to model the state transition of the enemy submarine, and the RFS-based measurement model to model the physical sensor of anti-submarine ship.

### 3.1. RFS-Based Data Model

Conventional estimation techniques fail to support digital twin for ASW, because many sophisticated simulation models of ASW cannot provide the analytical mathematical structures for deriving the functional forms of probability distribution. The sequential Monte Carlo (SMC) method which is also named as particle filter (PF), is the most widely used data-assimilation algorithm in recent years [29]. PF-based data-assimilation algorithm in traffic simulation is presented by Xu in [31] and Wu in [32]. PF-based data-assimilation algorithm in wildfire simulation is presented by Hu in [33,34]. They all use the same non-parametric statistic inference method based on PF, because PF has no assumptions on the distribution and linearity of the simulation model.

The conventional PF-based data-assimilation algorithm depends on the vector-based representation of data including states and measurements. The vector-based representation makes the vector-based data-assimilation algorithm have the following essential disadvantages:

1. It is based on the assumption that the studied system is a single dynamic system that is permanently active. It cannot be used for the dynamic system that switches on and off randomly. Switching is quite common for submarine activity, for example, a submarine may enter and leave a battle area at random instance.
2. It is based on the assumption that the detection is perfect with no false detections and no missed detections, and it also needs the number and ordering of measurements to be previously designated. Furthermore, it cannot jointly estimate the number of submarines and the states of each submarine.

Being different from the conventional vector-based representation, the RFS-based representation can take the more complex situations into consideration. The RFS-based representation of states can support the transition of submarines from one mode to another. RFS-based representation of states enables the submarine number to be constantly varying. For example, completely new submarines can enter a scene randomly. Submarines can likewise leave a scene, as when disappearing behind some other occlusion, or they can be damaged or destroyed. The RFS-based representation of measurements enables us to take the imperfection of the sensors into consideration. The sensors on the anti-submarine ship can fail to generate a measurement of the submarine state, or pick up false measurements. Since there is no information on which state generates the measurement, and the number of measurements is a random variable, RFS rather than vector-based representation can be more useful.

### 3.2. RFS-Based Measurement Model

The measurement model is in charge of mapping the simulation states to the measurements collected by the sensor. The sensor on the anti-submarine ship provides measurements with imperfect enemy submarine detection including noises, clutters, and missed detections. Detection uncertainty and clutters in the measurements can be described by the union of Bernoulli RFS and Poisson RFS as introduced in [35,36]. The sensor sequentially gives an unordered finite set of measurements $Z_{k}\subset Z$, and never indicates which of these measurements is generated from the enemy submarine.

The measurements produced by the sensor can be mathematically modeled as an RFS $Z=\{z_{1},z_{2},\ldots ,z_{m}\}$. Its major advantage is that both the measurements’ number m=|Z| and the value of the constituent vector z∈Z in the measurement space $Z\subseteq R^{n_{z}}$ are random. It makes no assumptions on the order of detections in the RFS Z. The measurements for ASW can be represented as Z=C∪W, here C is the Poisson random finite subset of false detections and W is the Bernoulli random finite subset corresponding to the enemy submarine.

The behavior of the sensor can be described by the conventional likelihood function g(z|x) which characterizes the probability that measurement *z* is generated by the submarine state x. Here we can have $g\left(z|x_{k}\right)=N\left(z;h\left(x_{k}\right),\omega_{k}\right)$. If a measurement $z\in Z_{k}$ is generated from an enemy submarine, then the relationship between $z\in Z_{k}$ and the submarine state $x_{k}$ can be described by following equation:

(1) $$z=h\left(x_{k}\right)+\omega_{k}$$

where $\omega_{k}$ is a zero-mean independent Gaussian noise with variance $\sigma_{\theta }^{2}$, and $h(x_{k})$ is in charge of modeling the true submarine bearing or range at time *k*. In the following part of this section, we will derive the specific mathematical form for the measurement model denoted by φ(Z|X), where X is the Bernoulli RFS of simulation states. The measurement model φ(Z|X) can be written in two specific forms, one for X=∅ and the other for X={x}.

Firstly, if the enemy submarine does not exist in the real operational area, the measurement set will only consist of clutters. It means that X=∅ and Z=C∪∅=C. Here we model the number of clutters in the measurement set by the following Poisson distribution:

(2) $$P\left\{|C|=s\right\}=\frac{e^{-\lambda }\lambda^{s}}{s!},s=0,1,2,\ldots$$

here λ is the average number of clutters. Clutters are modeled as independent identically distributed random vectors conditioned on |C|. The values of these random vectors are taken from the measurement space Z with probability density function (PDF) c(z). Here $c\left(z\right)={\left(2\pi \right)}^{-1}$ is the time invariant spatial distribution of clutters. The measurement model can be modeled as follows:

(3) $$\varphi \left(Z|\emptyset \right)=\kappa \left(Z\right)=e^{-\lambda }\prod_{z\in Z}\lambda c\left(z\right)$$

Secondly, if the enemy submarine exists in the real operational area with state X={x}. The measurement RFS W corresponding to the enemy submarine can be modeled as a Bernoulli RFS conditioned on X={x}. If the submarine fails to be detected, we have W=∅. If the submarine is detected and causes a measurement *z*, we have W={z}. To derive the measurement model φ(Z|{x}), we should specify the PDF of the RFS W conditioned on {x}. This PDF can be modeled as follows:

(4) $$\eta \left(W|\left\{x\right\}\right)=\left\{\begin{array}{cc} 1-p_{D}\left(x\right) & \mathrm{if}\;\;W=\emptyset \\ p_{D}\left(x\right)\cdot g\left(z|x\right), & \mathrm{if}\;\;W=\{z\}\\ 0, & \mathrm{otherwise} \end{array}\right.$$

where $p_{D}\left(x\right)$ is the probability of detecting the submarine state x.

The measurement model φ(Z|{x}) can be represented as $\varphi \left(Z|\left\{x\right\}\right)=\sum_{W\subseteq Z}\eta \left(W|\left\{x\right\}\right)\kappa \left(Z\setminus W\right)$, here ∖ is the set-difference operation and κ was defined by (3). Since the RFS W is a Bernoulli RFS, and the summation is computable, we can get the measurement model for the case X={x} as follows:

(5) $$\begin{array}{cc} \varphi (Z|\{x\}) & =\eta \left(\emptyset |\left\{x\right\}\right)\cdot \kappa \left(Z\right)+\sum_{z\in Z}\eta \left(\left\{z\right\}|\left\{x\right\}\right)\cdot \kappa \left(Z\setminus \left\{z\right\}\right) \end{array}$$

(6) $$\begin{array}{c} =\kappa \left(Z\right)\left[1-p_{D}\left(x\right)+p_{D}\left(x\right)\sum_{z\in Z}g\left(z|x\right)\frac{\kappa (Z\setminus \{z\})}{\kappa (Z)}\right] \end{array}$$

In summary, the RFS-based measurement model for ASW can be represented as follows:

(7) $$\varphi \left(Z|X\right)=\left\{\begin{array}{cc} \kappa (Z), & \mathrm{if}\;\;X=\emptyset \\ \kappa \left(Z\right)\left[1-p_{D}\left(x\right)+p_{D}\left(x\right)\sum_{z\in Z}g\left(z|x\right)\frac{\kappa (Z\setminus \{z\})}{\kappa (Z)}\right], & \mathrm{if}\;\;X=\{x\}\\ 0, & \mathrm{otherwise} \end{array}\right.$$

### 3.3. RFS-Based Simulation Model

Digital twin requires a simulation model that can predict possible future states. To give a unified description of the enemy submarine presence/absence in the operational area and its kinematic state, we employ the Bernoulli RFS-based simulation model to describe the dynamics of enemy submarine at discrete time *k*. The state space is ∅∪S(X), where S(X) is a set of all singleton {x} and x∈X. Here we use PDF to denote the uncertainty of the submarine’s states which are evolving according to the state space model in discrete time as follows:

(8) $$X_{k}=f_{k|k-1}\left(X_{k-1}\right)+v_{k}$$

here $f_{k|k-1}$ denotes the deterministic part of the true evolution equation which is in charge of mapping the state to the next time step, and v is the stochastic part of the true evolution that we fail to capture deterministically, and it makes assimilating online measurements necessary.

If the enemy submarine is existing in the real operational area, the state $X_{k}$ will be a singleton, and can be modeled by the Markov process whose Markov transition density is denoted by $\pi_{k|k-1}\left(x_{k}|x_{k-1}\right)$ during the simulation interval $T_{k}=t_{k}-t_{k-1}$. The submarine state vector is adopted as follows:

(9) $$x_{k}^{t}={\left[x_{k}^{t}\;\;{\dot{x}}_{k}^{t}\;\;y_{k}^{t}\;\;{\dot{y}}_{k}^{t}\right]}^{T}$$

where $\left(x_{k}^{t},y_{k}^{t}\right)$ and $\left({\dot{x}}_{k}^{t},{\dot{y}}_{k}^{t}\right)$ is respectively the enemy submarine position and velocity in Cartesian coordinate. The state vector of the anti-submarine ship $x_{k}^{o}$ is similarly represented by $x_{k}^{o}={\left[x_{k}^{o}\;\;{\dot{x}}_{k}^{o}\;\;y_{k}^{o}\;\;{\dot{y}}_{k}^{o}\right]}^{T}$. Here $\pi_{k|k-1}\left(x_{k}|x_{k-1}\right)$ is the nearly constant velocity (NCV) model and relies on the relative state vector which is defined as follows:

(10) $$x_{k}={\left[x_{k}\;\;{\dot{x}}_{k}\;\;y_{k}\;\;{\dot{y}}_{k}\right]}^{T}={\left[x_{k}^{t}-x_{k}^{o}\;\;{\dot{x}}_{k}^{t}-{\dot{x}}_{k}^{o}\;\;y_{k}^{t}-y_{k}^{o}\;\;{\dot{y}}_{k}^{t}-{\dot{y}}_{k}^{o}\right]}^{T}.$$

The specific mathematical form of $\pi_{k|k-1}\left(x_{k}|x_{k-1}\right)$ is described by the state space model as follows:

(11) $$x_{k+1}=F_{k}x_{k}-S_{k+1,k}+\varepsilon_{k}$$

here $F_{k}$ is the deterministic part of the true evolution equation, $S_{k+1,k}$ is a deterministic matrix related to the effect of anti-submarine ship acceleration, and $\varepsilon_{k}∼N\left(0,Q_{k}\right)$ is stochastic part of the true evolution equation. Here we have $\pi_{k|k-1}\left(x_{k}|x_{k-1}\right)=N\left(x;F_{k}x_{k-1}-S_{k+1,k},Q_{k}\right)$. Their specific forms are as follows:

(12) $$F_{k}=I_{2}⊗\left[\begin{array}{cc} 1 & T_{k}\\ 0 & 1 \end{array}\right],\;\;Q_{k}=I_{2}⊗\varpi \left[\begin{array}{cc} \frac{T_{k}^{3}}{3} & \frac{T_{k}^{2}}{2}\\ \frac{T_{k}^{2}}{2} & T_{k} \end{array}\right],\;\;S_{k+1,k}=\left[\begin{array}{c} x_{k+1}^{o}-x_{k}^{o}-T_{k}{\dot{x}}_{k}^{o}\\ {\dot{x}}_{k+1}^{o}-{\dot{x}}_{k}^{o}\\ y_{k+1}^{o}-y_{k}^{o}-T_{k}{\dot{y}}_{k}^{o}\\ {\dot{y}}_{k+1}^{o}-{\dot{y}}_{k}^{o} \end{array}\right]$$

where ⊗ is the Kronecker product, and ϖ is the intensity of process noise. Here we assume $T_{k}=T=const$, so we can get $F_{k}=F$ and $Q_{k}=Q$.

**Figure 3:**
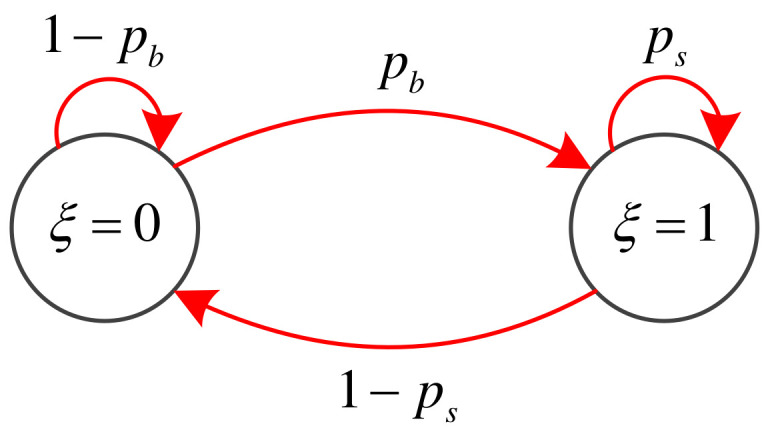
State transition diagram for Markov chain of the enemy submarine in ASW.

To characterize the enemy submarine’s appearance and disappearance during the simulation interval, we use a binary random variable $\xi_{k}\in \left\{0,1\right\}$ to denote its existence. Here $\xi_{k}=1$ indicates that the enemy submarine exists at time *k*, and $\xi_{k}=0$ indicates that it does not exist at time *k*. The dynamics of $\xi_{k}$ is described by the first-order 2-state Markov chain with a transitional probability matrix Ξ as it is shown in Figure 3. The elements of Ξ are defined as ${\left[\Xi \right]}_{ij}=P\left\{\xi_{k}=j-1|\xi_{k-1}=i-1\right\}$ for i,j∈{1,2}. Ξ is defined as follows:

(13) $$\Xi =\left[\begin{array}{cc} \left(1-p_{b}\right) & p_{b}\\ \left(1-p_{s}\right) & p_{s} \end{array}\right]$$

where $p_{b}=P\left\{\xi_{k+1}=1|\xi_{k}=0\right\}$ is the probability that the submarine appears in the operational area during the simulation interval, and $p_{s}=P\left\{\xi_{k+1}=1|\xi_{k}=1\right\}$ is the probability that the submarine is still in the operational area during the simulation interval. Here $p_{b}$ and $p_{s}$ are assumed to be known. If the submarine appears during the simulation interval $T_{k}$, PDF $b_{k|k-1}\left(x\right)$ can be used to denote its PDF.

Now we derive the RFS-based simulation model of enemy submarine. Since we represent the submarine’s simulation states by using Bernoulli RFS, we consider two different situations for the RFS-based simulation model.

Firstly, if the enemy submarine does not exist in the real operational area at time *k*, we can get $X_{k}=\emptyset$. In addition, the submarine can appear in the operational area with probability $p_{b,k}$ and have kinematic state $x_{k}$ with PDF $b_{k|k-1}\left(x_{k}\right)$, or remain absent from the operational area with probability $1-p_{b,k}$. The simulation model $f_{k|k-1}\left(X_{k}|\emptyset \right)$ for state $X_{k}$ is specified as follows:

(14) $$f_{k|k-1}\left(X_{k}|\emptyset \right)=\left\{\begin{array}{cc} 1-p_{b,k}, & \mathrm{if}\;\;X_{k}=\emptyset \\ p_{b,k}\cdot b_{k|k-1}\left(x_{k}\right), & \mathrm{if}\;\;X_{k}=\left\{x_{k}\right\}\\ 0, & \mathrm{otherwise} \end{array}\right.$$

Secondly, if the enemy submarine is existing in the real operational area at time k−1, which means $X_{k-1}=\left\{x_{k-1}\right\}$, it can come through to the next time step with probability $p_{s,k}\left(x_{k-1}\right)$ and transit to $x_{k}$ with PDF $\pi_{k|k-1}\left(x_{k}|x_{k-1}\right)$, or disappear with probability $1-p_{s,k}\left(x_{k-1}\right)$. Thus, the simulation model for state $X_{k}$ at time step *k* is characterized by

(15) $$f_{k|k-1}\left(X_{k}|\left\{x_{k-1}\right\}\right)=\left\{\begin{array}{cc} 1-p_{s,k}\left(x_{k-1}\right), & \mathrm{if}\;\;X_{k}=\emptyset \\ p_{s,k}\left(x_{k-1}\right)\cdot \pi_{k|k-1}\left(x_{k}|x_{k-1}\right), & \mathrm{if}\;\;X_{k}=\left\{x_{k}\right\}\\ 0, & \mathrm{otherwise} \end{array}\right.$$

From (14) and (15), we can know that the Bernoulli RFS can either be an empty set or a nonempty set with one element only. In addition, the probabilities of these two cases can be modeled respectively as 1−q and *q*. If it has one element, the element will be spatially distributed over $S\left(X\right)\subseteq R^{n_{x}}$ according to the standard PDF s(x). So, the simulation model for the simulation state denoted by the Bernoulli RFS $X_{k}$ at time *k* can be given by:

(16) $$f\left(X_{k}\right)=\left\{\begin{array}{cc} 1-q_{k|k}, & \mathrm{if}\;\;X_{k}=\emptyset \\ q_{k|k}\cdot s_{k|k}\left(x_{k}\right), & \mathrm{if}\;\;X_{k}=\left\{x_{k}\right\}\\ 0, & \mathrm{otherwise} \end{array}\right.$$

where $q_{k|k}=p\{|X_{k}\left|=1\right|Z_{1:k}\}$ is the probability of submarine’s existence in the real operational area, $s_{k|k}\left(x_{k}\right)=p\left(x_{k}|Z_{1:k}\right)$ is the spatial PDF of the submarine.

## 4. RFS-Based Data-Assimilation Algorithm

Unforeseen operational entities can enter the designated real operational area and critical assets can be destroyed, thereby invalidating the current simulation setting [37]. It is possible that the simulation execution may not proceed as expected because of the dynamic and changing environment. Since assimilating real-time measurements into the running simulation models can significantly improve the accuracy of the simulation results, the implementation of digital twin theory depends on the simulation system’s use of the information from the real world.

Here an RFS-based data-assimilation algorithm is proposed for online assimilating the sequence of measurement sets in the present of noise, false alarms, data association uncertainty, and detection uncertainty. The proposed RFS-based data-assimilation algorithm can overcome the limitations of the standard vector-based data-assimilation algorithms very well.

### 4.1. Data Assimilation with RFS-Based Models

Since data assimilation usually updates the posterior distribution of simulation states by using the simulation model and measurement model, it can be regarded as a Bayesian inference procedure from a probabilistic point of view [38]. Here we also use the RFS-based models and Bayesian inference for data assimilation.

The RFS-based data assimilation estimates recursively the posterior PDF of submarine’s states by using the RFS-based simulation model, measurement model, and online measurements. It usually consists of two stages, the prediction stage and the update stage. Since we have got a Bernoulli RFS-based simulation model (16), the posterior PDF at time *k*, denoted by $f_{k|k}\left(X_{k}|Z_{1:k}\right)$ is completely specified by two terms:

- the posterior submarine’s existence probability $q_{k|k}=p\{|X_{k}\left|=1\right|Z_{1:k}\}$;
- the posterior spatial PDF of $X_{k}=\left\{x\right\}$ denoted by $s_{k|k}\left(x_{k}\right)=p\left(x_{k}|Z_{1:k}\right)$.

Denote the posterior PDF of simulation states at time k−1 as $f_{k-1|k-1}\left(X_{k-1}|Z_{1:k-1}\right)$. The RFS-based prediction equation of the data-assimilation process is:

(17) $$\begin{array}{cc} f_{k|k-1}\left(X_{k}|Z_{1:k-1}\right) & =\int f_{k|k-1}\left(X_{k}|X^{{}^{\prime }}\right)f_{k-1|k-1}\left(X^{{}^{\prime }}|Z_{1:k-1}\right)\delta X^{{}^{\prime }} \end{array}$$

(18) $$\begin{array}{c} =f_{k|k-1}\left(X_{k}|\emptyset \right)f_{k-1|k-1}\left(\emptyset |Z_{1:k-1}\right)+\int f_{k-1|k-1}\left(\left\{x^{{}^{\prime }}\right\}|Z_{1:k-1}\right)dx^{{}^{\prime }}. \end{array}$$

At time k−1, the posterior PDF is characterized by the pair $\left(q_{k-1|k-1},s_{k-1|k-1}\left(x\right)\right)$, the prediction equation of data assimilation can be derived from (18) as:

(19) $$q_{k|k-1}=p_{b}\left(1-q_{k-1|k-1}\right)+p_{s}q_{k-1|k-1}$$

(20) $$s_{k|k-1}\left(x\right)=\frac{p_{b}\left(1-q_{k-1|k-1}\right)b_{k|k-1}\left(x\right)+p_{s}q_{k-1|k-1}\int \pi_{k|k-1}\left(x|x^{{}^{\prime }}\right)s_{k-1|k-1}\left(x^{{}^{\prime }}\right)dx^{{}^{\prime }}}{p_{b}\left(1-q_{k-1|k-1}\right)+p_{s}q_{k-1|k-1}}$$

In Bayesian theory, the updated PDF is calculated by:

(21) $$f_{k|k}\left(X_{k}|Z_{1:k}\right)=\frac{\varphi_{k}\left(Z_{k}|X_{k}\right)f_{k|k-1}\left(X_{k}|Z_{1:k-1}\right)}{\int \varphi_{k}\left(Z_{k}|X\right)f_{k|k-1}\left(X|Z_{1:k-1}\right)\delta X}=\frac{\varphi_{k}\left(Z_{k}|X_{k}\right)f_{k|k-1}\left(X_{k}|Z_{1:k-1}\right)}{f_{k}\left(Z_{k}|Z_{1:k-1}\right)}$$

Given the measurement model (7) and the prediction Equations (19) and (20), the update equation can be derived from (21) as:

(22) $$q_{k|k}=\frac{1-\delta_{k}}{1-\delta_{k}\cdot q_{k|k-1}}\cdot q_{k|k-1}$$

(23) $$s_{k|k}\left(x\right)=\frac{1-p_{D}+p_{D}\sum_{z\in Z_{k}}\frac{g_{k}\left(z|x\right)}{\lambda c(z)}}{1-\delta_{k}}\cdot s_{k|k-1}\left(x\right)$$

where

(24) $$\delta_{k}=p_{D}\left(1-\sum_{z\in Z_{k}}\frac{\int g_{k}\left(z|x\right)s_{k|k-1}\left(x\right)dx}{\lambda c(z)}\right).$$

### 4.2. SMC-Based Calculation

In the general cases, the RFS-based prediction equation and update equation cannot be solved analytically [39]. Here we propose a SMC-based calculation. We use the particle system ${\left\{w_{k}^{\left(i\right)},x_{k}^{\left(i\right)}\right\}}_{i=1}^{N}$ to approximate the spatial PDF $s_{k|k}\left(x\right)$. Here $x_{k}^{\left(i\right)}$ is the state of particle *i* and $w_{k}^{\left(i\right)}$ is its weight. As $s_{k|k}\left(x\right)$ is a conventional PDF, the corresponding weights of the particles should be normalized, i.e., $\sum_{i=1}^{N}w_{k}^{\left(i\right)}=1$.

**Figure 4:**
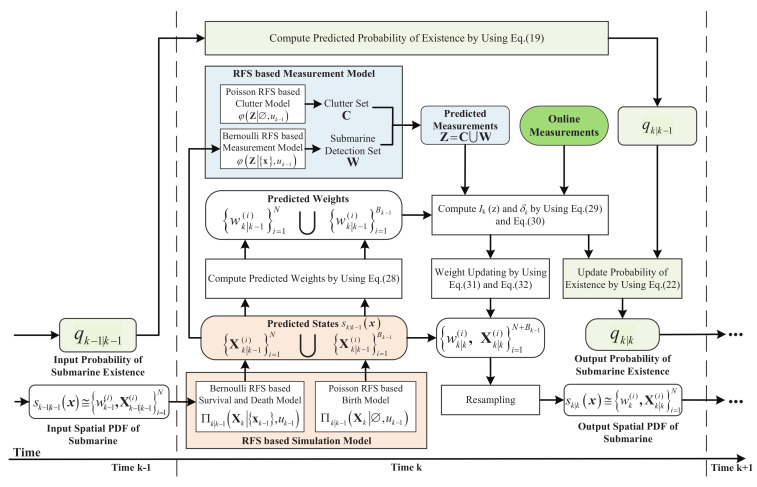
Schematic Diagram of the SMC-Based Calculation of the RFS-Based Data Assimilation.

As it is shown in Figure 4, the RFS-based simulation model firstly runs to the next time point and generates the predicted states of ASW. Weight updating relies on the difference between the online measurements and the predicted measurements generated from the predicted states. Suppose at time k−1, the submarine’s existence probability is $q_{k-1|k-1}$, and the spatial PDF is approximated by

(25) $${\hat{s}}_{k-1|k-1}\left(x\right)=\sum_{i=1}^{N}w_{k-1}^{\left(i\right)}\delta_{x_{k-1}^{\left(i\right)}}\left(x\right).$$

here $\delta_{c}\left(x\right)$ is the Dirac delta function concentrated at state c. The predicted submarine’s existence probability $q_{k|k-1}$ can be computed by (19). According to (20) and (25), the predicted spatial PDF depends on the sum of two terms. Consequently $s_{k|k-1}\left(x\right)$ can be approximated by the particle system as a weighted point mass representation:

(26) $${\hat{s}}_{k|k-1}\left(x\right)=\sum_{i=1}^{N+B_{k}}w_{k|k-1}^{\left(i\right)}\delta_{x_{k|k-1}^{\left(i\right)}}\left(x\right).$$

here the particles are drawn from two importance densities, $\rho_{k}$ for persisting particles and $\beta_{k}$ for birth particles:

(27) $$x_{k|k-1}^{\left(i\right)}∼\left\{\begin{array}{cc} \rho_{k}\left(x|x_{k-1}^{\left(i\right)},Z_{k}\right) & i=1,\ldots ,N\\ \beta_{k}\left(x|Z_{k}\right) & i=N+1,\ldots ,N+B_{k} \end{array}\right.$$

with weights

(28) $$w_{k|k-1}^{\left(i\right)}=\left\{\begin{array}{cc} \frac{p_{s}\cdot q_{k-1|k-1}}{q_{k|k-1}}\frac{\pi_{k|k-1}\left(x_{k}^{\left(i\right)}|x_{k-1}^{\left(i\right)}\right)}{\rho_{k}\left(x_{k|k-1}^{\left(i\right)}|x_{k-1}^{\left(i\right)},Z_{k}\right)}w_{k-1}^{\left(i\right)}, & i=1,\ldots ,N\\ \frac{p_{b}\cdot \left(1-q_{k-1|k-1}\right)}{q_{k|k-1}}\frac{b_{k|k-1}\left(x_{k|k-1}^{\left(i\right)}\right)}{\beta_{k}\left(x_{k|k-1}^{\left(i\right)}|Z_{k}\right)}\frac{1}{B_{k}}, & i=N+1,\ldots ,N+B_{k}. \end{array}\right.$$

where $B_{k}$ is the number of submarine-birth particles drawn from the importance density $\beta_{k}$.

The simplest choice of importance density $\rho_{k}\left(x|x_{k-1},Z_{k}\right)$ is the transitional density $\pi_{k|k-1}\left(x_{k}|x_{k-1}\right)$. If there is little prior knowledge of the action plan of enemy submarine, we should assume that the enemy submarine can appear anywhere in the state space S(X). So, we model $b_{k|k-1}\left(x\right)$ by using the uniform distribution over S(X). The birth importance density $\beta_{k}$ in (27) needs to have the same support as $b_{k|k-1}\left(x\right)$ (i.e., the entire S(X)) [40].

The update step of the data-assimilation algorithm is implemented according to (22)–(24). First, for every $z\in Z_{k}$, the integral $I_{k}\left(z\right)=\int g_{k}\left(z|x\right)s_{k|k-1}\left(x\right)dx$, which appears in (24), is approximately calculated as follows:

(29) $$I_{k}\left(z\right)\approx \sum_{i=1}^{N+B_{k}}w_{k|k-1}^{\left(i\right)}\cdot g_{k}\left(z|x_{k|k-1}^{\left(i\right)}\right)$$

Then based on (24), $\delta_{k}$ can be calculated by

(30) $$\delta_{k}\approx p_{D}\left(1-\sum_{z\in Z_{k}}\frac{I_{k}\left(z\right)}{\lambda c(z)}\right).$$

The submarine’s existence probability is updated by using (22), and the corresponding weights are updated according to (23):

(31) $${\tilde{w}}_{k|k}^{\left(i\right)}\approx \left[1-p_{D}+p_{D}\sum_{z\in Z_{k}}\frac{g_{k}\left(z|x_{k|k-1}^{\left(i\right)}\right)}{\lambda c(z)}\right]\cdot w_{k|k-1}^{\left(i\right)}$$

These weights should be normalized to get the normalized importance weights:

(32) $$w_{k|k}^{\left(i\right)}=\frac{{\tilde{w}}_{k|k}^{\left(i\right)}}{\sum_{j=1}^{N+B_{k}}{\tilde{w}}_{k|k}^{\left(j\right)}}$$

for $i=1,\ldots ,N+B_{k}$. At last, we resample *N* times from ${\left\{w_{k|k}^{\left(i\right)},x_{k|k-1}^{\left(i\right)}\right\}}_{i=1}^{N+B_{k}}$ to avoid sample degeneracy.

## 5. Computation of Reward Function

We propose an online sensor control method by using the predicted states, updated states, and their corresponding weights generated by the proposed data-assimilation algorithm. Online sensor control is mainly used for finding the optimal control action from a set of admissible control actions. Thus, it means sequential decision making, where each decision generates measurements that provide an additional information for data assimilation. In the digital twin for ASW, the control action is determined in the present of uncertainty both in the measurement space and the state space. Here an information theoretic approach is proposed for online sensor control. In this approach, the posterior PDF is used to represent the uncertain states, and the reward function is regarded as a measure of the information gain related to each action.

### 5.1. Derivation of Reward Function

In this paper, the online sensor control means the online selection of headings for individual anti-submarine ship, to maximize the use efficiency of its measurement system. Here control actions are ranked by using the quantity of information predicted to be gained from their execution. The data-assimilation enhanced simulation model is used to rapidly predict the possible output information of alternative control actions.

Reward function is used to measure the reduction in the information gain, in comparison with the current information state. The information gain can be characterized by using various information measures [41]. The Fisher information is typically used as a criterion for optimization in the absence of detection uncertainty [42,43,44]. Here we use the Rényi divergence-based reward function. The Rényi information divergence provides a way to measure the dissimilarity between two probability densities [45]. The Rényi divergence between any two probability densities $p_{0}\left(x\right)$ and $p_{1}\left(x\right)$ is described as:

(33) $$I_{\alpha }\left(p_{1},p_{0}\right)=\frac{1}{\alpha -1}\log\int p_{1}^{\alpha }\left(x\right)p_{0}^{1-\alpha }\left(x\right)dx$$

where α≥0 is the factor that reflects how much we emphasize the tails of two probability distributions.

Let $u_{k}\in U_{k}$ denote the control action chosen for controlling the sensor at time $t_{k}$ in order to collect the future measurements at $t_{k+1}$. Here $U_{k}$ denotes the set of admissible control actions at time $t_{k}$. In general, both the simulation model $f_{k+1|k}$ and the measurement model $\varphi_{k+1}$ depend on the control action $u_{k}\in U_{k}$. Then the prediction Equation (18) and update Equation (21) for RFS-based data assimilation can be rewritten as follows:

(34) $$f_{k+1|k}\left(X_{k+1}|Z_{1:k},u_{0:k}\right)=\int f_{k+1|k}\left(X|X^{{}^{\prime }},u_{k}\right)f_{k|k}\left(X^{{}^{\prime }}|Z_{1:k},u_{0:k-1}\right)\delta X^{{}^{\prime }}$$

(35) $$f_{k+1|k+1}\left(X_{k+1}|Z_{1:k+1},u_{0:k}\right)=\frac{\varphi_{k+1}\left(Z_{k+1}|X_{k+1},u_{k}\right)f_{k+1|k}\left(X_{k+1}|Z_{1:k},u_{0:k}\right)}{\int \varphi_{k}\left(Z_{k+1}|X,u_{k}\right)f_{k+1|k}\left(X|Z_{1:k},u_{0:k}\right)\delta X}$$

The optimal control action to be applied at time *k* is defined by maximizing the expected Rényi information divergence according to equation

(36) $$u_{k}=\arg\max_{v\in U_{k}}E\left\{\phi (v,p\left(X_{k+1}|Z_{1:k},u_{1:k-1}\right),Z_{k+1}\left(v\right))\right\}$$

where ϕ(v,p,Z) is the real-valued reward function associated with the control action v. (36) results in the predicted PDF *p* and the future measurement set Z. Online sensor control via (36) tries to obtain the maximum reward based on a single future step. This is done by anticipating possible future measurements. To find the optimal control action for the anti-submarine ship to take next, we should predict the system states if the control action $u_{k}$ has been chosen by using the RFS-based simulation model. In addition, we should generate the predicted measurement set before actually receiving the measurement set $Z_{k+1}$. Hence the calculation of the expected value of the Rényi divergence for each possible control action is closely related to the data-assimilation process.

The future measurement set $Z_{k+1}\left(v\right)$ supports the computation of the reward function ϕ. Since $Z_{k+1}\left(v\right)$ is obtained after the control action has been executed, this will create uncertainty. To overcome the impact of uncertainty, (36) employs the expectation operator E. The reward function $\phi (u_{k},p,Z)$ in (36) is adopted as the Rényi divergence between:

- the predicted PDF $f_{k+1|k}\left(X_{k+1}|Z_{1:k},u_{0:k}\right)$ given by (34) which is based on action $u_{k}$, and
- the predicted future posterior $f_{k+1|k+1}\left(X_{k+1}|Z_{1:k+1},u_{0:k}\right)$ given by (35), obtained by using the new measurement set $Z_{k+1}$ collected after the sensor has been controlled to take action $u_{k}$.

We simplify the reward function ϕ by suppressing its second and third argument. Depending on (33), the reward function ϕ can be represented by:

(37) $$\phi \left(u_{k}\right)=\frac{1}{\alpha -1}\log\int {\left[f_{k+1|k+1}\left(X_{k+1}|Z_{1:k+1},u_{0:k}\right)\right]}^{\alpha }{\left[f_{k+1|k}\left(X_{k+1}|Z_{1:k},u_{0:k}\right)\right]}^{1-\alpha }\delta X_{k+1}$$

### 5.2. Data-Assimilation-Based Computation

The expected reward function $E[\phi \left(u_{k}\right)]$ does not have the closed-form analytic solution. Thus, we employ numerical approximate method and data-assimilation-based solution for it. This makes the SMC-based implementation of data assimilation become valuable. By adopting the SMC-based implementation, the optimal control action selection can be implemented quickly. Furthermore, this makes the data-assimilation process and online sensor control to be interdependent and interoperable.

Since the predicted PDF and the updated PDF in the RFS-based data-assimilation process are Bernoulli PDFs, let $f_{k+1|k}\left(X_{k+1}|Z_{1:k},u_{0:k}\right)$ and $f_{k+1|k+1}\left(X_{k+1}|Z_{1:k+1},u_{0:k}\right)$, which feature in (37), be specified by the pairs ($q_{k+1|k}$, $s_{k+1|k}\left(x\right)$) and ($q_{k+1|k+1}$, $s_{k+1|k+1}\left(x\right)$) respectively. So, the predicted PDF and the updated PDF can be written as follows:

(38) $$f_{k+1|k}\left(X_{k+1}|Z_{1:k},u_{0:k}\right)=\left\{\begin{array}{cc} 1-q_{k+1|k} & \mathrm{if}\;X_{k+1}=\emptyset ,\\ q_{k+1|k}\cdot s_{k+1|k}\left(x\right) & \mathrm{if}\;X_{k+1}=\left\{x\right\},\\ 0 & \mathrm{otherwise}. \end{array}\right.$$

(39) $$f_{k+1|k+1}\left(X_{k+1}|Z_{1:k+1},u_{0:k}\right)=\left\{\begin{array}{cc} 1-q_{k+1|k+1} & \mathrm{if}\;X_{k+1}=\emptyset ,\\ q_{k+1|k+1}\cdot s_{k+1|k+1}\left(x\right) & \mathrm{if}\;X_{k+1}=\left\{x\right\},\\ 0 & \mathrm{otherwise}. \end{array}\right.$$

According to the rules of set integral, the reward function defined in (37) can be simplified to:

(40) $$\begin{array}{cc} \phi (u_{k})= & \frac{1}{\alpha -1}\log\{{\left[1-q_{k+1|k}\right]}^{1-\alpha }{\left[1-q_{k+1|k+1}\right]}^{\alpha }\\ & +{\left[q_{k+1|k}\right]}^{1-\alpha }{\left[q_{k+1|k+1}\right]}^{\alpha }\int {\left[s_{k+1|k}\left(x\right)\right]}^{1-\alpha }\cdot {\left[s_{k+1|k+1}\left(x\right)\right]}^{\alpha }dx\} \end{array}$$

According to (36), the optimal control action can be selected as the expected value:

(41) $$u_{k}=\arg\max_{v\in U_{k}}E\left\{\phi \left(v\right)\right\}$$

Now we use SMC to obtain the numerical implementation of (41). First, we obtain the values of the reward functions for the predicted future measurement set sequence $Z_{k+1}\left(v\right)$. Then we compute the expected value of ϕ(v) by calculating the sample mean of the obtained values. Here $Z_{k+1}\left(v\right)$ is obtained after taking control action $v\in U_{k}$. Each realization of $Z_{k+1}\left(v\right)$ is generated from the predicted PDF represented by $\left(q_{k+1|k},s_{k+1|k}\left(x\right)\right)$ by using the RFS-based measurement model.

Basing on the SMC-based implementation of the proposed data-assimilation algorithm, we select *M* predicted submarine states from ${\left\{w_{k+1|k}^{\left(i\right)},x_{k+1|k}^{\left(i\right)}\right\}}_{i=1}^{N+B_{k}}$ with probability $w_{k+1|k}^{\left(i\right)}$. So, we can get *M* predict ideal measurements for the computation of the reward function. The key of computing (40) is the computation of the following integral

(42) $$Q=\int {\left[s_{k+1|k}\left(x\right)\right]}^{1-\alpha }\cdot {\left[s_{k+1|k+1}\left(x\right)\right]}^{\alpha }dx$$

Depending on the SMC-based implementation of the proposed data assimilation, it can be computed as follows. Let $s_{k+1|k}\left(x\right)$ be approximated by ${\left\{w_{k+1|k}^{\left(i\right)},x_{k+1|k}^{\left(i\right)}\right\}}_{i=1}^{N+B_{k}}$. By taking control action v, we can obtain a sample of the future measurement set ${\left\{Z_{k+1}^{\left(i\right)}\left(v\right)\right\}}_{i=1}^{M}$ with *M* submarine originated noiseless measurement set. According to the proposed RFS-based data-assimilation algorithm, $s_{k+1|k+1}\left(x\right)$ can be approximated by the particle system ${\left\{w_{k+1|k+1}^{\left(i\right)},x_{k+1|k+1}^{\left(i\right)}\right\}}_{i=1}^{N+B_{k}}$, where $w_{k+1|k+1}^{\left(i\right)}$ is computed according to (31)–(32). Equation (42) relies on the measurement set ${\left\{Z_{k+1}^{\left(i\right)}\left(v\right)\right\}}_{i=1}^{M}$ and $w_{k+1|k+1}^{\left(i\right)}$, and is approximated by:

(43) $$Q\approx \sum_{i=1}^{N+B_{k}}{\left(w_{k+1|k}^{\left(i\right)}\right)}^{1-\alpha }\cdot {\left(w_{k+1|k+1}^{\left(i\right)}\right)}^{\alpha }$$

In conclusion, the computation of the reward function assigned to every control action $v\in U_{k}$ for the RFS-based online sensor control method is as follows:

(44) $$\begin{array}{cc} \phi (v)= & \frac{1}{\alpha -1}\log\{{\left[1-q_{k+1|k}\right]}^{1-\alpha }{\left[1-q_{k+1|k+1}\right]}^{\alpha }\\ & +{\left[q_{k+1|k}\right]}^{1-\alpha }{\left[q_{k+1|k+1}\right]}^{\alpha }\sum_{i=1}^{N+B_{k}}{\left(w_{k+1|k}^{\left(i\right)}\right)}^{1-\alpha }\cdot {\left(w_{k+1|k+1}^{\left(i\right)}\right)}^{\alpha }\} \end{array}$$

followed by the application of (41).

## 6. Simulation Experiments

To verify the effectiveness of the proposed digital twin and RFS-based approach for sensor control in ASW, we have carried out three groups of experiments: the first one is related to the data-assimilation algorithm, the second one is related to the online sensor control with single submarine, the last one is related for the online sensor control with multiple submarines. The data-assimilation experiment is used to verify the correctness and effectiveness of the proposed RFS-based data-assimilation algorithm for assimilating the online measurements to the simulation system. The following two groups of online sensor control experiments are-based the data-assimilation experiment, and used to verify the proposed sensor control method for ASW. They use the prediction results of the data-assimilation experiment to control the real sensor of the anti-submarine ship.

### 6.1. Data-Assimilation Experiment

**Figure 5:**
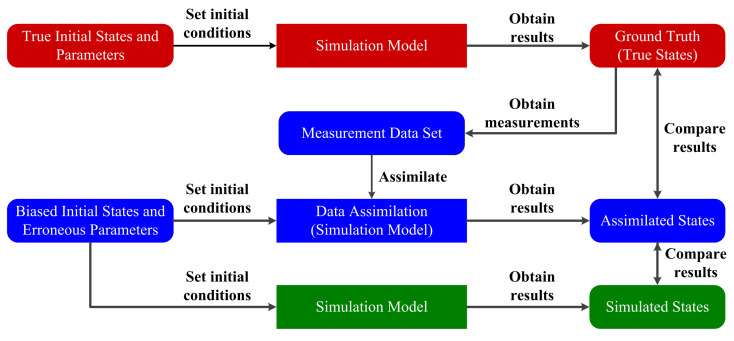
Identical-twin experiment procedure.

As it is shown in Figure 5, we adopt the identical-twin experiment to evaluate the proposed data-assimilation algorithm [34,46]. In this experiment, the RFS-based simulation model of enemy submarine with designated initial setting is first running, and the measurements corresponding to the simulation results are recorded. These simulation results are regarded as the real states of the physical ASW. And the measurements recorded here are regarded as the real-time measurements generated by the real sensor. We assimilate the measurements by using the proposed data-assimilation algorithm, and then compare the assimilated simulation states with the obtained real states.

As it is shown in Figure 5, we use three terms in the experiment: the real submarine state, the assimilated one, and the simulated one, to present the experimental result. A real submarine state is the simulated one from which the measurements are recorded. To reflect the fact that the submarine simulation execution usually depends on the biased initial parameters as compared with the real submarine in ASW, here the simulated submarine state is the simulation result based on some biased initial parameters, for example, imprecise process noise intensity. Here “biased” means in the sense that the parameters are different from those used in the real submarine state. Finally, an assimilated submarine state is the data-assimilation enhanced simulation result based on the same biased initial parameters as in the simulated one. The goal of this experiment is to prove that the assimilated submarine state is more accurate than the simulated one by assimilating measurements.

#### 6.1.1. Experimental Setup

In the real ASW, the submarine moves at a speed of approximately 5 knots. The scan repetition time of the sensor on the anti-submarine ship is 30 s. The probability of detection is assumed to be Gaussian distributed with mean 0 and covariance $\sigma_{D}=5000$. The number of clutters per scan is assumed to be Poisson distributed with the mean value λ=1. The parameters of the data-assimilation process are as follows: particle number N=5000, birth probability $p_{b}=0.01$. The initial parameters for the enemy submarine and the anti-submarine ship are presented in Table 1.

The performance measure of experimental results is the positional root mean square (RMS) error defined as follows:

(45) $$\varepsilon_{k}=\sqrt{\frac{1}{P}\sum_{p=1}^{P}\left[{\left({\hat{x}}_{k|k}^{\left(p\right)}-x_{k}\right)}^{2}+{\left({\hat{y}}_{k|k}^{\left(p\right)}-y_{k}\right)}^{2}\right]}$$

where *P* is the total number of Monte Carlo runs, $\left({\hat{x}}_{k|k}^{\left(p\right)},{\hat{y}}_{k|k}^{\left(p\right)}\right)$ is the assimilated (or simulated) submarine state at time *k* in the *p*th run, and $\left(x_{k},y_{k}\right)$ is the ground truth.

#### 6.1.2. Experimental Results

**Figure 6:**
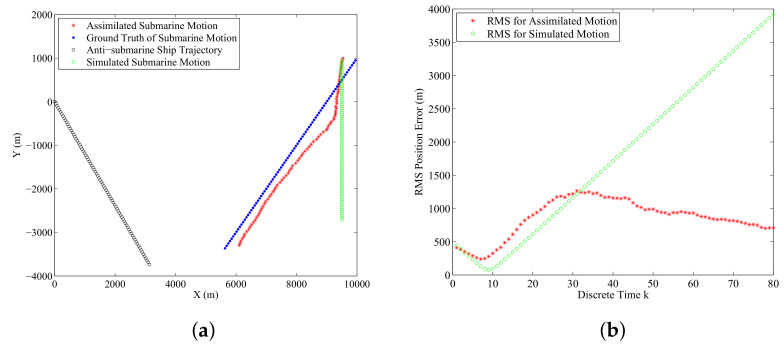
The experimental results averaged over 500 Monte Carlo simulations: (**a**) Anti-submarine ship motion, simulated submarine trajectory and the assimilated one; (**b**) Error performance of the assimilated submarine state and the simulated one.

Figure 6a displays the real submarine trajectory, simulated one, and assimilated one by averaging over 500 Monte Carlo runs. Figure 6b displays the RMS error curves by averaging 500 Monte Carlo runs. This experiment tests the effectiveness of the proposed data-assimilation algorithm when the process noise intensity, initial speed, heading, and position are biased. From Figure 6 we can see that the simulated one has large deviations from the real submarine state because of the erroneous initial parameters. However, the assimilated submarine state is much closer to the real one. By using the proposed data-assimilation algorithm, the assimilated submarine state overcomes the problem of erroneous initial parameters, and matches the real submarine state with much smaller errors.

**Figure 7:**
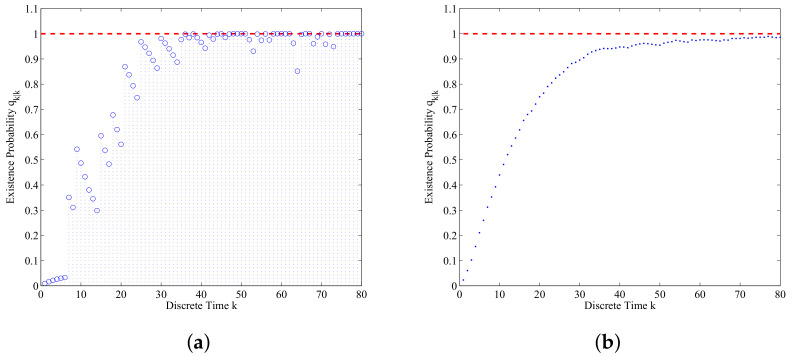
The submarine’s existence probability $q_{k|k}$ versus k: (**a**) The assimilated submarine’s existence probability of a single run; (**b**) The assimilated submarine’s existence probability averaged over 500 Monte Carlo simulations.

Figure 7a illustrates a typical result of a single run of the submarine’s existence probability obtained by the data-assimilation algorithm with ϖ=0.2. The red dotted line $q_{k|k}=1$ is the ground truth of the submarine’s existence probability which means that the enemy submarine exists in the real ASW all the way. The submarine’s existence probability shown in Figure 7a grows to 1 after some time steps and remains high throughout the simulation execution. If the detection of the submarine is missing, it drops but is still bigger than 0.8. Figure 7b shows the submarine’s existence probability averaged over 500 Monte Carlo simulations. We can find that as time involves, the assimilated submarine’s existence probability gradually approaches to 1. The occasional missed detections and false detections could not affect markedly the performance of the data-assimilation algorithm for this application.

The results of data-assimilation experiment prove that the proposed data-assimilation algorithm can successfully assimilate the online measurements to the running simulation model of ASW. In the following section, we will analyze the sensitivity of the proposed data-assimilation algorithm.

#### 6.1.3. Sensitivity Analysis

**Figure 8:**
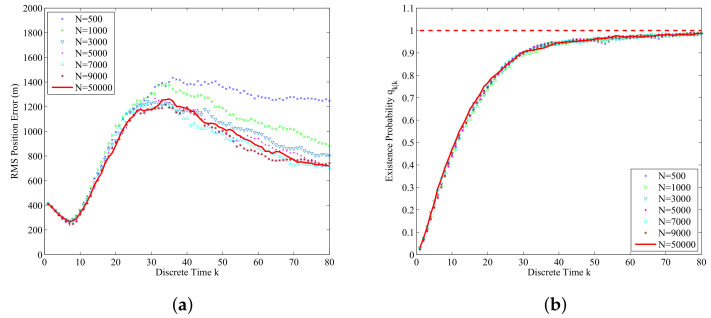
The data-assimilation results for N= 500, 1000, 3000, 5000, 7000, 9000 and 50,000 (averaged over 500 Monte Carlo simulations): (**a**) RMS position errors for different *N*; (**b**) The assimilated probability of submarine existence for different *N*.

The influence of particle number *N* on the overall performance of the proposed data-assimilation algorithm is studied by using different particle numbers. Figure 8a shows the RMS position errors averaged over 500 Monte Carlo simulations for different particle number *N*. From Figure 8a we can find that if the particle number *N* increases, the RMS position error will decrease. However, if the particle number *N* is larger than a certain degree, the influence of the particle number *N* on the RMS position error will be every small. This is consistent with standard particle filter theory. Figure 8b shows the influence of the particle number *N* on the estimated probability of submarine existence $q_{k|k}$ for k=1,2,…,80. From Figure 8b we can find that the influence of particle number *N* on the estimated probability of submarine existence is very limited for this application.

**Figure 9:**
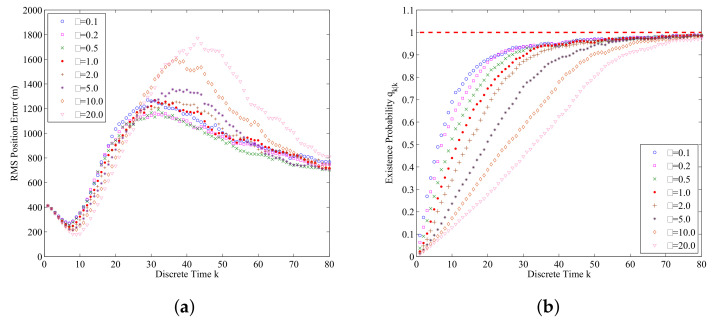
The data-assimilation results for λ=0.10, 0.20, 0.50, 1.0, 2.0, 5.0, 10.0 and 20.0 (averaged over 500 Monte Carlo simulations): (**a**) RMS position errors for different λ; (**b**) The assimilated probability of submarine existence for different λ.

To estimate the influence of the mean value λ of clutters on the performance of the data-assimilation algorithm, Figure 9a,b shows the RMS position errors and assimilated probability of submarine existence curves obtained from different λ. From Figure 9a we can find that if λ is small than a certain value, its influence on RMS position error is very limited. However, if λ increases, the RMS position error will gradually increase, too. Figure 9b shows that if the mean value λ of clutters increases, the error of estimated probability of submarine existence will also increase. In addition, it will take more time for assimilated probability of submarine existence to approach the ground truth. The results agree with Equation (22), (23) and (31). When λ increases, the updated weight *w* for the particles representing the true submarine states will decrease, this leads to the increasing of RMS position error.

**Figure 10:**
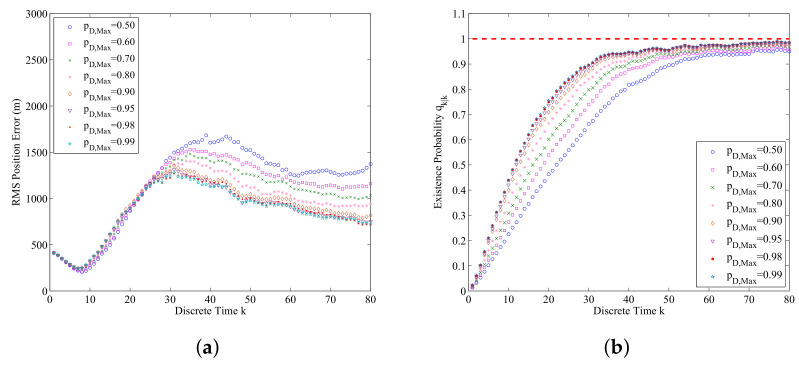
The data-assimilation results for $p_{D,Max}=$0.50, 0.60, 0.70, 0.80, 0.90, 0.95, 0.98, and 0.99 (averaged over 500 Monte Carlo simulations): (**a**) RMS position error for different $p_{D,Max}$; (**b**) The estimated probability of submarine existence for different $p_{D,Max}$.

We also compare the performance of the data-assimilation algorithm for the different settings of max detection probability $p_{D,Max}$. The results are as shown in Figure 10a,b. From these figures, we can find that the bigger $p_{D,Max}$ is, the smaller the errors of RMS position and probability of submarine existence are. If $p_{D,Max}$ increases, according to (31), the updated weights for the submarine generated measurements will increase. This also makes the accuracy of data-assimilation increase.

**Figure 11:**
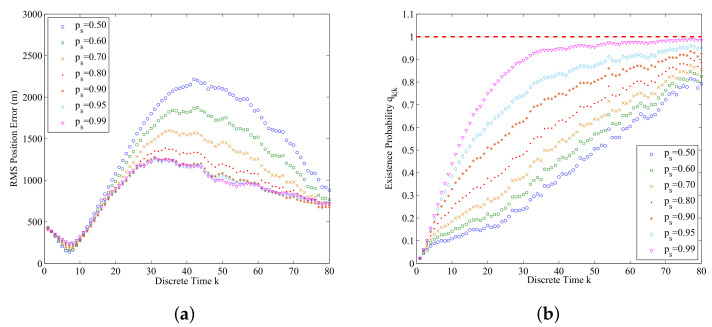
The data-assimilation results for $p_{s}=$0.50, 0.60, 0.70, 0.80, 0.90, 0.95, and 0.99 (averaged over 500 Monte Carlo simulations): (**a**) RMS position errors for different $p_{s}$; (**b**) The assimilated probability of submarine existence for different $p_{s}$.

The influence of $p_{s}$ on the performance of the data-assimilation algorithm is shown in Figure 11a,b. From Figure 11a,b we can find that the increase of $p_{s}$ will improve the accuracy of estimated submarine states and probability of submarine existence. From Equation (19), we know that if $p_{s}$ increases, the predicted existence probability *q* will also increase. From Equation (28), we know that if $p_{s}$ increases, the predicted weights for the survival particles will increase, and this leads to the decreasing of the RMS position error.

### 6.2. Online Sensor Control Experiment with Single Submarine

To verify the effectiveness of the proposed online sensor control method, we use a scenario where the anti-submarine ship trajectory consists of only two constant velocity motion legs. The online sensor control is conducted at the end of the first leg, when the choice is between different turns at different headings. In this experiment, there is only one enemy submarine in the operational area.

#### 6.2.1. Experimental Setup

**Figure 12:**
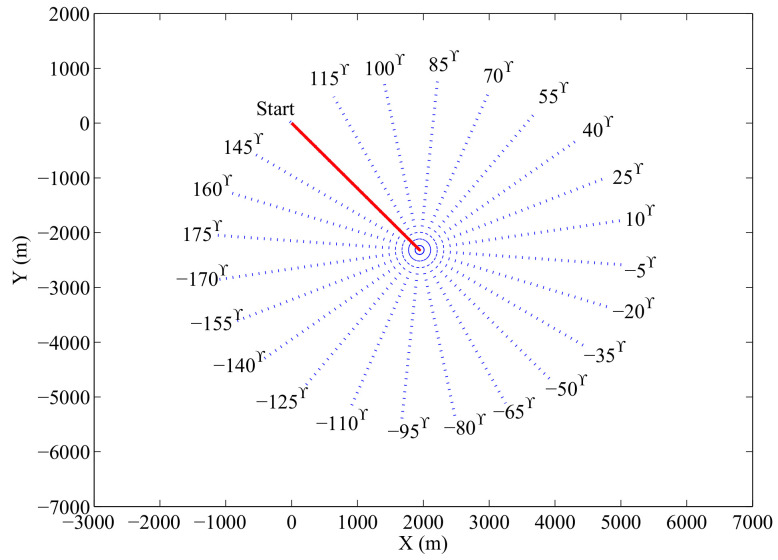
The set of admissible future legs of the anti-submarine ship trajectory.

During the first leg, the speed of the anti-submarine ship is 4 knots and the course during this leg is $-50^{\circ }$. As it is shown in Figure 12, at the end of the first leg (k=50), the anti-submarine ship needs to choose a new course for the second leg. We verify the performance of the proposed online sensor control method by using detection parameters $p_{D,Max}=0.98$, $\sigma_{D}=5000$ and λ=5. To find the best option for the anti-submarine ship heading among the 24 second leg options, we need to obtain the RMS position error at time step k=51 for each admissible course $\theta =-170^{\circ },100^{\circ },\ldots ,175^{\circ }$. We obtained these RMS position errors by fixing the value of θ and conducting 500 Monte Carlo runs for each θ to compute the averaged RMS position errors.

The set of admissible control actions $U_{k}$ is determined as follows. If the current position of the anti-submarine ship is $u_{k}={\left[\chi_{k}\;\;\psi_{k}\right]}^{T}$, its future admissible locations are:

(46) $$U_{k}=\left\{\left(\chi_{k}+V_{ship}\cdot \cos\left(l{▵}_{\theta }+\theta_{0}\right),\psi_{k}+V_{ship}\cdot \sin\left(l{▵}_{\theta }+\theta_{0}\right)\right);\;\;l=1,\ldots ,N_{\theta }\right\}$$

where ${▵}_{\theta }=2\pi /N_{\theta }$ is a selected course step size, $\theta_{0}=-50^{\circ }$ is the initial course of the anti-submarine ship, $N_{\theta }=24$, and $V_{ship}=$ 4 knots. The anti-submarine ship can move in its current course (case l=24) or move in other courses. 24 control actions are considered at the end of the first motion leg.

We first get the RMS position errors for all the courses at the time step at which the control actions are executed. Then we test the online sensor control method by comparing the number of times out of 500 Monte Carlo runs that it has chosen for each particular course.

#### 6.2.2. Experimental Results

**Figure 13:**
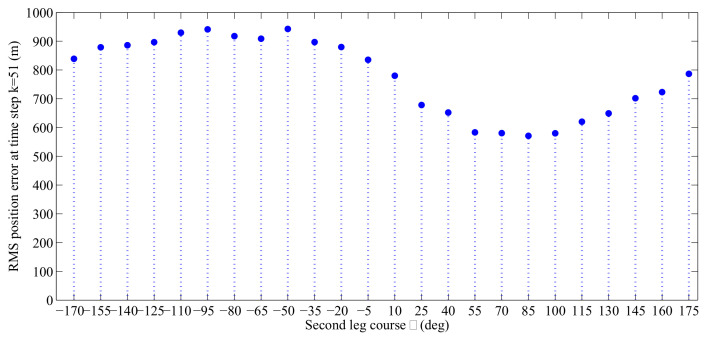
RMS positional error obtained by averaging over 500 Monte Carlo runs at time step k = 51 for all admissible second leg anti-submarine ship course $\theta =-170^{\circ },-155^{\circ },\ldots ,175^{\circ }$.

**Figure 14:**
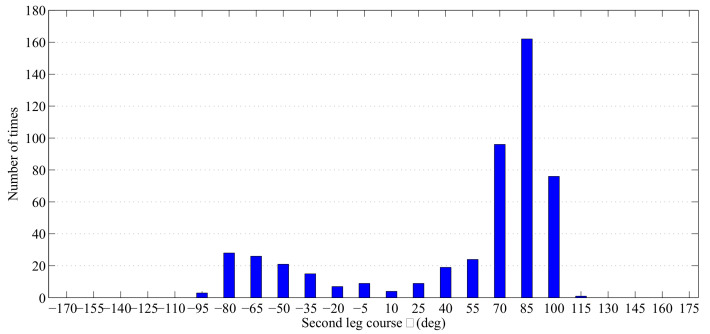
Number of times (out of 500 Monte Carlo runs) a particular second leg anti-submarine ship courses θ has been chosen for parameter α=0.8, M=20.

The RMS position errors for different courses are plotted in Figure 13. It indicates that the second leg course decisions of $70^{\circ }$ and $85^{\circ }$ are preferred in this experiment. After finding out the best control actions for the anti-submarine ship’s second leg heading, we let the anti-submarine ship make its own decision by using the proposed online sensor control method. Figure 14 shows the number of times out of 500 Monte Carlo runs the online sensor control method has chosen for each particular course. From Figure 13 and Figure 14, we can know that the proposed online sensor control method can give a suitable control decision by using the digital twin-based framework of sensor control in ASW.

The results successfully verify the correctness and effectiveness of the proposed digital twin and RFS-based framework. In the following section, we will study the influence of the number *M* of prediction measurements on the results.

#### 6.2.3. Sensitivity Analysis

We analyze the influence of the number *M* of prediction measurements on the proposed online sensor control method. Table 2 shows the results for different values of *M*. From Table 2, we know that when *M* increases, the number of times for the good control actions (including courses $70^{\circ }$ and $85^{\circ }$) increases, and the number of times for the bad control actions decreases. However, the influence extent is very limited, since *M* increases quickly and the performance of the method is slightly improved.

### 6.3. Online Sensor Control Experiment with Multiple Submarines

In this experiment, we verify the effectiveness of the proposed online sensor control method by a scenario where the anti-submarine ship tracks multiple submarines. Here we control the range-only sensor of the anti-submarine ship by using the proposed digital twin and RFS-based method.

#### 6.3.1. Experimental Setup

The state of single submarine at time step *k* is represented by $x_{k}={\left[p_{k}^{T}\;\;v_{k}^{T}\right]}^{T}$, here $p_{k}={\left[x_{k}\;\;y_{k}\right]}^{T}$ is the position, $v_{k}={\left[v_{k,x}\;\;v_{k,y}\right]}^{T}$ is the velocity. The position of the anti-submarine ship is represented by $u_{k}^{o}={\left[x_{k}^{o}\;\;y_{k}^{o}\right]}^{T}$. The probability of detecting the enemy submarine at position $p_{k}={\left[x_{k}\;\;y_{k}\right]}^{T}$ is computed as follows:

(47) $$p_{D}\left(p_{k}\right)=\left\{\begin{array}{cc} 1, & \mathrm{if}\;\;∥p_{k}-u_{k}^{o}∥\leq R_{0}\\ \max\{0,1-(∥p_{k}-u_{k}^{o}∥-R_{0})\hbar \}, & \mathrm{if}\;\;∥p_{k}-u_{k}^{o}∥>R_{0} \end{array}\right.$$

here $∥p_{k}-u_{k}^{o}∥=\sqrt{{\left(x_{k}-x_{k}^{o}\right)}^{2}+{\left(y_{k}-y_{k}^{o}\right)}^{2}}$ is the distance between the sensor and the submarine at $p_{k}$. In this experiment, the operational area is a square of sides s=1200 m, $R_{0}=300$ and ℏ=0.0002 m${}^{-1}$. The measurement is generated by $z=h\left(x_{k}\right)=∥p_{k}-u_{k}^{o}∥+\omega$, where ω is zero-mean white Gaussian measurement noise, with deviation $\sigma_{\omega }=\sigma_{0}+\beta {∥p_{k}-u_{k}^{o}∥}^{2}$. In this experiment, $\sigma_{0}=1$ m and $\beta =5\;\times \;10^{-5}$ m${}^{-1}$. The clutters are modeled as the Poisson RFS. The intensity of clutters is modeled by the uniform densityκ(z)=λ·c(z) with mean λ=5.

In this experiment, there are 5 moving enemy submarines in the operational area. As it is shown in (46), we control the sensor by finding the optional course. To prove the validity of the proposed method, we compare the proposed control method with the other method that the control vector is randomly selected from the set $U_{k}$. Each method runs 10 times, and the averaged OSPA errors (order parameter p=2 and cutoff c=100 m) are compared.

#### 6.3.2. Experimental Results

**Figure 15:**
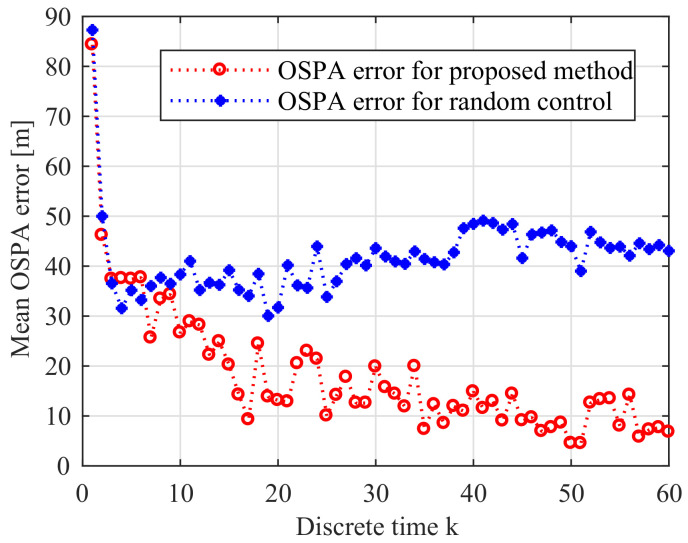
Error performance of two methods of 10 Monte Carlo simulations.

In this experiment, the anti-submarine ship controls the sensor and runs the sensor control method every $T_{c}=5$ time steps. We compare the performance by using the OSPA error at every time step. The mean OSPA errors of two methods are given in Figure 15. We can see that the proposed digital twin and RFS-based online sensor control method can effectively reduce the OSPA error. We can also find that as time evolves, the OSPA error of the proposed method also gradually reduces in this application.

**Figure 16:**
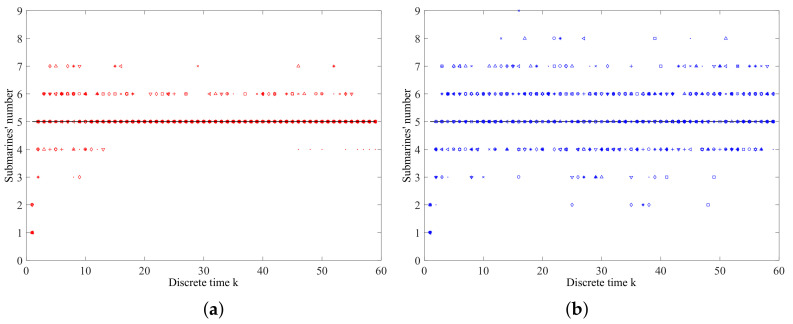
The estimated submarines’ number for two methods of 10 Monte Carlo simulations: (**a**) Estimated submarines’ number by the proposed sensor control method; (**b**) Estimated submarines’ number by the random control method.

Figure 16 gives the estimated submarines’ numbers of 10 Monte Carlo simulations generated by using the proposed data-assimilation algorithm and the different online sensor control methods. The black line represents the truth of submarines’ number, and the data points of different shapes represent the estimated submarines’ number at each time step of 10 Monte Carlo simulations. Figure 16a is the result of the proposed sensor control method, and Figure 16b is the result of the random control method. We can find that the proposed sensor control method performs much better than the random control method on estimating submarines’ number.

**Figure 17:**
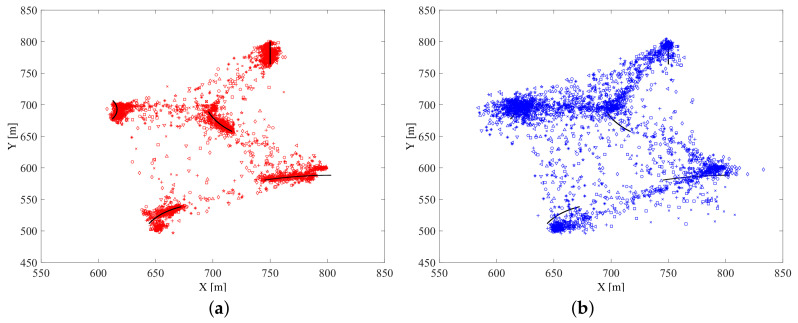
The estimated submarines’ states for two methods of 10 Monte Carlo simulations: (**a**) Estimated submarines’ states by the proposed sensor control method; (**b**) Estimated submarines’ states by the random control method.

Figure 17 gives the estimated submarines’ states of 10 Monte Carlo simulations generated by using the proposed data-assimilation algorithm and the different online sensor control methods. The black lines represent the truth of submarines’ states, and the data points of different shapes represent the truth of the submarines’ states. Figure 17a is the result of the proposed sensor control method, and Figure 17b is the result of the random control method. We can find that the proposed sensor control method also performs much better than the random control method for estimating submarines’ states.

**Figure 18:**
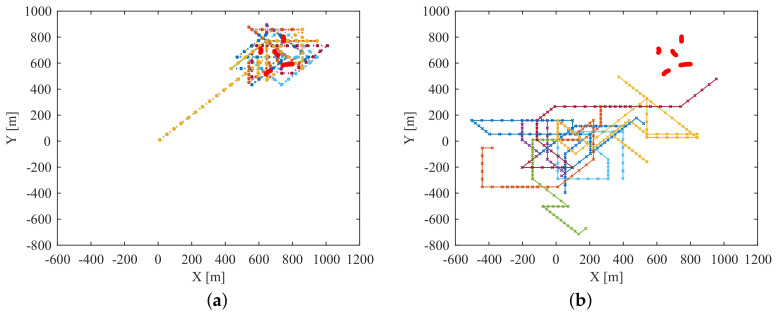
The paths of the anti-submarine ship of 10 Monte Carlo simulations for two methods: (**a**) Paths of anti-submarine ship controlled by the proposed sensor control method; (**b**) Paths of anti-submarine ship controlled by the random control method.

The paths of the anti-submarine ship for two methods are shown in Figure 18. Different colors represent different Monte Carlo simulations, and the red points represent the truth of the submarines’ trajectories. We can see that the proposed sensor control method can successfully guide the sensor to move close to the enemy submarines to get more accurate and more reliable measurements.

#### 6.3.3. Sensitivity Analysis

In this section, the performance of the prosed online sensor control method is further analyzed by sensitivity analysis. We analyze some parameters’ influence on the performance of the proposed sensor control method. These parameters are the time interval $T_{c}$ of two sensor control actions, factor α, the number *M* of prediction measurements, and particle number *N* for each submarine.

**Figure 19:**
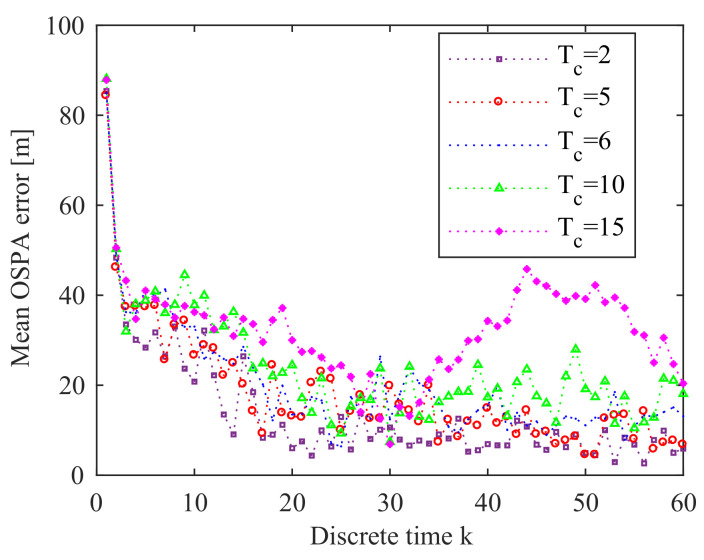
Error performance for $T_{c}$ = 2, 5, 6, 10, and 15 (averaged over 10 Monte Carlo simulations).

**Figure 20:**
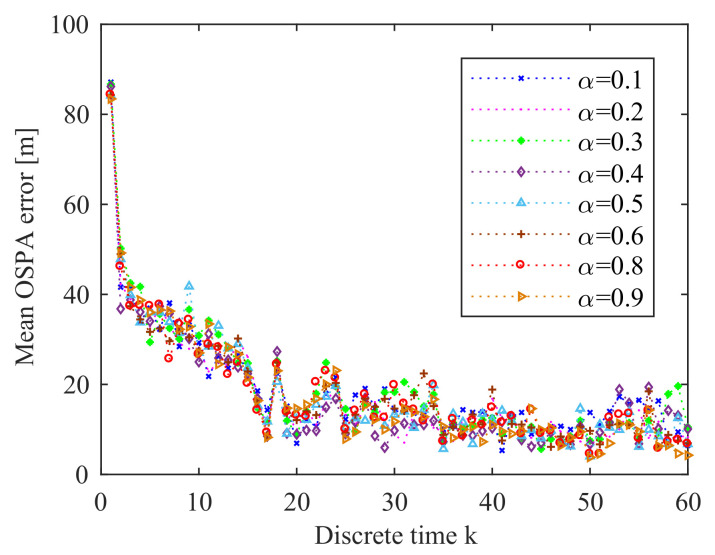
Error performance for α = 0.1, 0.2, 0.3, 0.4, 0.5, 0.6, 0.8, and 0.9 (averaged over 10 Monte Carlo simulations).

**Figure 21:**
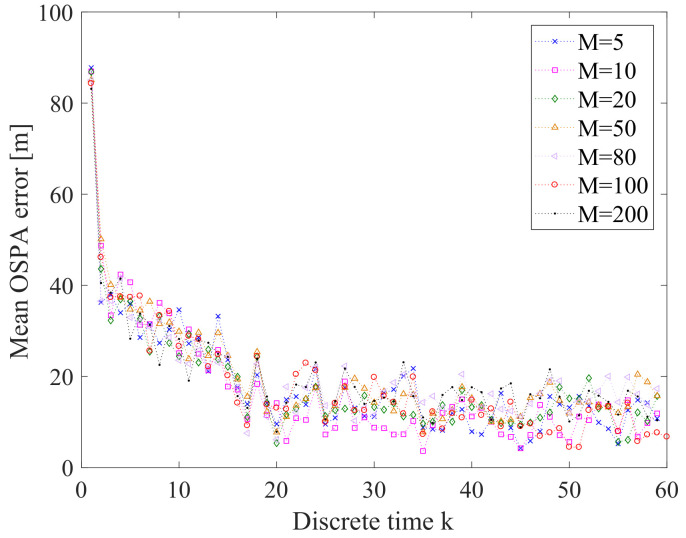
Error performance for *M* = 5, 10, 20, 50, 80, 100 and 200 (averaged over 10 Monte Carlo simulations).

**Figure 22:**
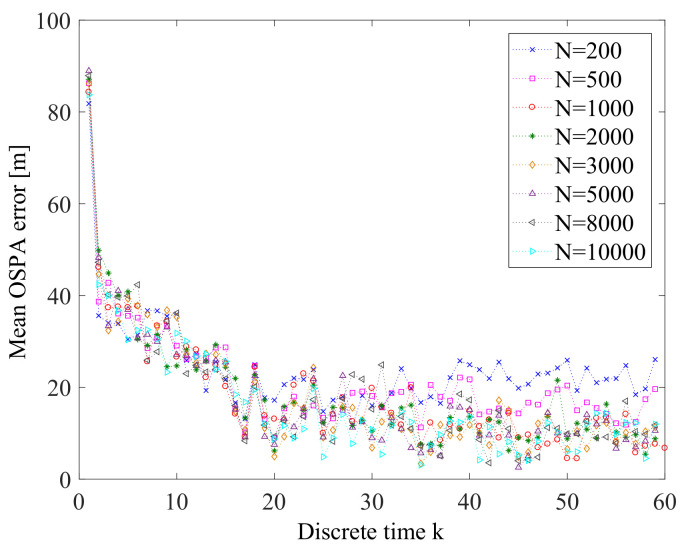
Error performance for *N* = 200, 500, 1000, 2000, 3000, 5000, 8000, and 10,000 (averaged over 10 Monte Carlo simulations).

From Figure 19, we can see that as $T_{c}$ increases, the mean OSPA error also increases. This means that decreasing $T_{c}$ can improve the performance of the prosed online sensor control method. Figure 20 gives the OSPA error for various values of parameter α. We can find that α has quite little influence on the performance of the proposed sensor control method in this application. The influence of the number *M* of prediction measurements on the online sensor control method is shown in Figure 21. We can see that the influence of *M* on the performance of the proposed sensor control method is little. The reason is that the performance of the proposed method does not only depend on the reward function, but also depends on the data-assimilation algorithm. As it is shown in Figure 22, the particle number *N* can affect the performance of the proposed method. The increase of *N* can ensure more reasonable choice of the control action.

## 7. Conclusions

In this paper, we studied the digital twin-based framework of sensor control in ASW. We firstly combine the simulated ASW with the real ASW by employing the digital twin theory. Then we proposed an RFS-based data-assimilation algorithm to dynamically incorporate online measurements generated from the real ASW. At last, we also fostered the ability of the simulation system to control the sensor in ASW by deriving and implementing the data-assimilation-based reward function. The proposed data-assimilation algorithm has the potential to overcome the limitations of the conventional vector-based algorithms. It can jointly estimate the number of enemy submarines and the state of each enemy submarine. We tested the proposed data-assimilation algorithm by using the identical-twin experiment. The results prove that the proposed algorithm can assimilate the input measurements and improve the accuracy of simulation results. We tested the proposed online sensor control method with two group of experiments, including the scenario with single submarine and the scenario with multiple submarines. The results showed that the proposed online sensor control method can effectively control the sensor. This paper can be regarded as an application of digital twin in ASW and the methods can also be applied to other applications.

## Figures and Tables

**Table 1:** Parameters settings in the experiment.

Name	Parameter	True Value	Biased Value
Enemy submarine	Initial speed	5 knot	3 knot
	Process noise intensity ϖ	0.0	0.2
	Initial position	(10,000, 1000) m	(9500, 1000) m
	Initial heading	−135 deg	−90 deg
	Survival Probability $p_{s}$	0.99	-
Anti-submarine ship	Initial speed	4 knot	-
	Initial heading	−50 deg	-
	Initial position	(0, 0) m	-
	Detection probability $P_{D}$	N(x,0,5000)	-
	Max detection probability $p_{D,Max}$	0.98	-
	Measurement standard deviation $\sigma_{\theta }$	1 deg	-

**Table 2:** Number of times (out of 500 Monte Carlo runs) a particular second leg course θ has been chosen for different values of parameter *M*.

θ∖M	1	5	10	20	50	80	100	200	500	800	900	1000	1500	2000
$-170^{\circ }$	0	0	0	0	0	0	0	0	0	0	0	0	0	0
$-155^{\circ }$	0	0	0	0	0	0	0	0	0	0	0	0	0	0
$-140^{\circ }$	1	0	0	0	0	0	0	0	0	0	0	0	0	0
$-125^{\circ }$	0	0	0	0	0	0	0	0	0	0	0	0	0	0
$-110^{\circ }$	8	0	0	0	0	0	0	0	0	0	0	0	0	0
$-95^{\circ }$	25	11	11	3	1	0	0	0	0	0	0	0	0	0
$-80^{\circ }$	45	23	37	28	22	15	17	11	9	5	7	6	8	2
$-65^{\circ }$	45	42	27	25	32	30	32	33	30	29	30	30	31	32
$-50^{\circ }$	22	30	29	22	20	16	20	19	19	26	18	15	20	17
$-35^{\circ }$	19	17	14	15	8	12	10	7	12	12	12	10	8	12
$-20^{\circ }$	2	7	10	7	7	8	6	8	6	4	5	8	6	5
$-5^{\circ }$	7	9	5	9	6	6	6	8	6	6	5	4	5	4
$10^{\circ }$	4	3	6	4	3	5	3	2	3	6	1	7	4	7
$25^{\circ }$	9	12	9	9	9	8	6	4	5	4	4	3	6	5
$40^{\circ }$	18	10	12	19	14	13	13	9	11	9	16	13	12	11
$55^{\circ }$	26	30	28	24	39	32	32	35	30	34	39	28	26	22
$70^{\circ }$	65	90	82	96	108	120	110	108	104	102	92	106	94	91
$85^{\circ }$	111	125	142	162	175	192	207	225	242	256	263	266	276	290
$100^{\circ }$	64	77	81	76	56	43	38	31	23	7	8	4	4	2
$115^{\circ }$	22	13	7	1	0	0	0	0	0	0	0	0	0	0
$130^{\circ }$	5	1	0	0	0	0	0	0	0	0	0	0	0	0
$145^{\circ }$	1	0	0	0	0	0	0	0	0	0	0	0	0	0
$160^{\circ }$	0	0	0	0	0	0	0	0	0	0	0	0	0	0
$175^{\circ }$	1	0	0	0	0	0	0	0	0	0	0	0	0	0

## Abbreviations

The following abbreviations are used in this manuscript:

ASW	Anti-submarine Warfare
FISST	Finite Set Statistics
PDF	Probability Density Function
PF	Particle Filter
POMDP	Partially Observed Markov Decision Process
RFS	Random Finite Set
RMS	Root Mean Square
SMC	Sequential Monte Carlo

## References

[1] Jauffret C., Pillon D. (1996). Observability in passive target motion analysis. IEEE Trans. Aerosp. Electron. Syst..

[2] Nardone S.C., Aidala V.J. (1981). Observability criteria for bearing-only target motion analysis. IEEE Trans. Aerosp. Electron. Syst..

[3] Fogel E., Gavish M. (1988). Nth-order dynamics target observability from angle measurements. IEEE Trans. Aerosp. Electron. Syst..

[4] Doucet A., Vo B.-N., Andrieu C., Navy M. Particle filtering for multi-target tracking and sensor management. Proceedings of the 5th Annual Conference on Information Fussion (FUSION 2002).

[5] Aughenbaugh J.M., La Cour B.R. Metric selection for information theoretic sensor management. Proceedings of the 2008 11th International Conference on Information Fusion.

[6] Mahler R. (1998). Global posterior densities for sensor management. Proc. SPIE.

[7] Mahler R., Grundel D., Murphey R., Pardalos P. (2004). Multitarget sensor management of dispersed mobile sensors. Theory and Algorithms for Cooperative Systems.

[8] El-Fallah A., Zatezalo A., Mahler R., Donatelli D. (2008). Dynamic sensor management of dispersed and disparate sensors for tracking resident space objects. Proc. SPIE.

[9] Witkoskie J., Kuklinski W., Theophanis S., Otero M. Random set tracker experimen on a road constrained network with resource management. Proceedings of the 2006 9th International Conference on Information Fusion.

[10] Jenssen R., Principe J.C., Erdognus D., Eltoft T. (2006). The Cauchy-Schwarz divergence and Parzen windowing: connections to graph theory and Mercer kernels. J. Frankl. Inst..

[11] Ristic B., Vo B. (2014). Sensor control for multi-object state-space estimation using random finite sets. Automatica.

[12] Hoang H.G., Vo B.T. (2014). Sensor management for multi-target tracking via multi-Bernoulli filtering. Automatica.

[13] Hoang H.G., Vo B.N., Vo B.T., Mahler R. (2015). The Cauchy-Schwarz divergence for Poinsson point processes. IEEE Trans. Inf. Theory.

[14] Beard M., Vo B., Vo B., Arulanpalam S. Sensor Control for Multi-target Tracking using Cauchy-Schwarz Divergenc. Proceedings of the 2015 18th International Conference on Information Fusion (Fusion).

[15] Gostar A.K., Hoseinnezhad R., Rathnayake T., Wang X., Bab-Hadiashar A. (2017). Constrained sensor control for Labeled Multi-Bernoulli filter using Cauchy-Schwarz divergenc. IEEE Signal Process. Lett..

[16] Gomes-Borges M.E., Maltese D., Vanheeghe P., Duflos E. A risk-based sensor management using random finite sets and POMDP. Proceedings of the 2017 20th International Conference on Information Fusion (Fusion).

[17] Castanon D.A., Carin L., Hero A.O., Castanon D.A., Cochran D., Kastella K. (2008). Stochastic control theory for sensor management. Foundations and Applications of Sensor Management.

[18] Qi Q., Zhao D., Liao T.W., Tao F. Modeling of Cyber-physical Systems and Digital Twin Based on Edge Computing, Fog Computing and Cloud Computing Towards Smart Manufacturing. Proceedings of the ASME 2018 13th International Manufacturing Science and Engineering Conference.

[19] Grieves M., Vickers J., Kahlen F-JFlumerfelt S., Alves A. (2017). Digital Twin: Mitigating Unpredictable, Undesir-able Emergent Behavior in Complex Systems. Transdisciplinary Perspectives on Complex Systems: New Findings and Approaches.

[20] Abramovici M., Gobel J.C., Dang H.B. (2016). Semantic data management for the development and continuous reconfiguration of smart products and systems. Cirp Ann. Manuf. Technol..

[21] Rosen R., Von Wichert G., Lo G., Bettenhausen K.D. (2015). About the importance of autonomy and digital twins for the future of manufacturing. IFAC-PaperOnLine.

[22] Schlus M., Rossmann J. From simulation to experimentable digital twins: Simulation-based development and operation of complex technical systems. Proceedings of the 2016 IEEE International Symposium on Systems Engineering (ISSE).

[23] Schroeder G.N., Steinmetz C., Pereira C.E., Espindola D.B. (2016). Digital twin data modeling with automationML and a communication methodology for data exchange. Int. Fed. Autom. Control..

[24] Hochhalter J.D., Leser W.P., Newman J.A., Glaessgen E.H., Gupta V.K., Yamakov V.I. Coupling Damage-Sensing Particles to the Digitial Twin Concept. https://ntrs.nasa.gov/search.jsp?R=20140006408.

[25] Glaessgen E.H., Stargel D.S. The Digital Twin Paradigm for Future NASA and U.S. Air Force Vehicles. Proceedings of the 53rd AIAA Structures, Structural Dynamics and Materials Conference.

[26] Boschert S., Christoph H., Rosen R., Horvath J.P., Suarez Rivero P.M., Castellano H. (2018). Next Generation Digital Twin. Proceedings of TMCE 2018.

[27] Rao V., Sandu A. (2014). A posteriori error estimates for DDDAS inference problems. Procedia Comput. Sci..

[28] GE The Digital Twin: Compressing Time-to-Value for Digital Industrial Companies, White Paper. https://www.ge.com/digital/sites/default/files/The-Digital-Twin-Compressing-Time-to-Value-for-Digital-Industrial-Companies.pdf.

[29] Hu X. (2011). Dynamic data driven simulation. Scs Model. Simul. Mag..

[30] Douglas C.C. (2014). An Open Framework for Dynamic Big-Data-Driven Application Systems (DBDDAS) Development. Procedia Comput. Sci..

[31] Xie X., Verbraeck A., Gu F. Data Assimilation in Discrete Event Simulations—A Rollback based Sequential Monte Carlo Approach. Proceedings of the 2016 Spring Simulation Conference (SpringSim 2016).

[32] Wu P., Xue H., Hu X. Particle Filter Based Traffic Data Assimilation with Sensor Informed Proposal Distribution. Proceedings of the 2015 Spring Simulation Conference (SpringSim 2015).

[33] Xue H., Gu F., Hu X. (2012). Data Assimilation Using Sequential Monte Carlo Methods in Wildfire Spread Simulation. Acm Trans. Model. Comput. Simul..

[34] Xue H., Hu X. An Effective Proposal Distribution for Sequential Monte Carlo Methods-Based Wildfire Data Assimilation. Proceedings of the 2013 Winter Simulations Conference (WSC).

[35] Vo B.T., Vo B.-N., Cantoni A. (2008). Bayesian filtering with random finite set observations. IEEE Trans. Signal Process..

[36] Blackman S.S., Popoli R. (1999). Design and Analysis of Modern Tracking Systems.

[37] La Scala B.F., Mallick M., Arulampalam S. (2007). Differential geometry measures of nonlinearity for filtering with nonlinear dynamic and linear measurement models. SPIE Proc..

[38] Chen W., Kesidis G., Morrison T., Tinsley J., Fujimoto R., Bock C., Chen W., Page E., Panchal J.H. (2017). Uncertainty in Modeling and Simulation. Research Challenges in Modeling and Simulation for Engineering Complex Systems.

[39] van Leeuwen P.J., Cheng Y., Reich S., Jones C.K.R.T., Young L.-S. (2010). Nolinear data assimilation for high-dimensional sytems. Nonlinear Data Assimilation.

[40] Vo B.-T., Vo D.C.B.-N., Ristic B. (2011). Bernoulli forward-backward smoothing for joint target detection and tracking. IEEE Trans. Signal Proces..

[41] Hero A.O., Kreucher C.M., Blatt D., Hero A.O., Castanon D., Cochran D., Kastella K. (2008). Information theoretic approaches to sensor management. Foundations and Applications of Sensor Management.

[42] Passerieux J.M., Cappel D.V. (1998). Optimal observer maneuver for bearings-only tracking. IEEE Trans. Aerosp. Electr. Syst..

[43] Cadre J.-P.L., Laurent-Michel S. (1999). Optimizing the receiver maneuvers for bearings-only tracking. Automatica.

[44] Oshman Y., Davidson P. (1999). Optimization of observer trajectories for bearings only target localization. IEEE Trans. Aerosp. Electron. Syst..

[45] Kreucher C.M., Hero A.O., Kastella K.D., Morelande M.R. (2007). An information based approach to sensor management in large dynamic networks. Proc. IEEE.

[46] Nakamura K., Yamamoto S., Honda M. Sequential Data Assimilation in Geotechnical Engineering and Its Application to Seepage Analysis. Proceedings of the 14th International Conference on Information Fusion.

